# Managing conflicting ethical concerns in modern small animal practice—A comparative study of veterinarian’s decision ethics in Austria, Denmark and the UK

**DOI:** 10.1371/journal.pone.0253420

**Published:** 2021-06-18

**Authors:** Svenja Springer, Peter Sandøe, Herwig Grimm, Sandra A. Corr, Annemarie T. Kristensen, Thomas Bøker Lund

**Affiliations:** 1 Unit of Ethics and Human-Animal Studies, Messerli Research Institute, University of Veterinary Medicine Vienna, Medical University of Vienna, University of Vienna, Vienna, Austria; 2 Department of Food and Resource Economics, University of Copenhagen, Frederiksberg, Denmark; 3 Department of Veterinary and Animal Science, University of Copenhagen, Frederiksberg, Denmark; 4 Division of Small Animal Clinical Sciences, School of Veterinary Medicine, University of Glasgow, Glasgow, Scotland; 5 Department of Veterinary Clinical Science, Faculty of Health and Medical Sciences, University of Copenhagen, Frederiksberg, Denmark; Newcastle University, School of Natural and Environmental Sciences, UNITED KINGDOM

## Abstract

Small animal veterinarians frequently have to manage conflicting interests. Beside the key consideration of the patient’s interests, small animal veterinarians are often challenged to consider not only client’s emotional needs, but also their own personal aspirations to provide quality patient care and to make a good living as a professional. Further, veterinarians have an interest in continuous professional development and the use of the newest treatments, which may influence their decision-making process. Based on published work, we hypothesize the existence of four decision ethics orientations that veterinarians can use to manage potentially conflicting concerns. These are: the patient-focused, the client-empathetic, the client-devolved and the development-oriented decision ethics orientations. We surveyed small animal veterinarians in Austria, Denmark, and the UK using a questionnaire (N = 648), and successfully identified the four decision ethics orientations in all three countries. The patient-focused and client-empathetic decision ethics orientations are salient in all countries, whereas Danish and UK veterinarians are slightly more client-empathetic and client-devolved compared to their Austrian colleagues. Across countries our findings show that experienced and older veterinarians tend to be more client-empathetic. Younger and less experienced professionals are more development-oriented compared to their older and more experienced colleagues. In contrast to other studies investigating ethical issues in small animal practice, we found no evidence that gender plays a decisive role in the tendency towards any decision ethics orientation. We also show that veterinarians with a higher client-empathetic orientation and development-orientation more often discuss the possibility of health insurance with clients who do not have it. The present study provides a first empirical insight into how veterinarians manage challenging expectations and ethical concerns as part of decision making in modern small animal practice.

## Introduction

Patient care is a key focus for the veterinary profession, but recent research has revealed that small animal veterinarians (from here on veterinarians) are increasingly challenged by trying to balance concerns for the patient, the client, and the veterinarian personally. Empirical studies indicate that strong emotional relationships between the client and the animal [1–7], the client’s financial resources [8–11], the veterinarian’s own economic interests and working background including level of specialization and/or practice type [1] as well as time available for consultation [12–14] all impact veterinarian’s work life and can lead to potential ethical concerns and challenges in clinical decision making.

Clients’ financial limitations are one of the most challenging aspects of providing veterinary care to a standard that most veterinarians would consider as best practice [8–11]. In a focus group study comprising Austrian veterinarians, Springer and colleagues [1] found that the use of advanced diagnostic and therapeutic methods is often associated with higher costs, which can create a multi-tier health care system. This can lead to ethical and financial concerns for the veterinarians’ themselves. While the implementation of modern technologies in the clinic provides an opportunity to improve patient care, the purchase of new equipment also leads to economic concerns, as the equipment must pay for itself, while being affordable for the client.

In addition, the development of specialized skills and the implementation of new techniques enables veterinarians to offer diagnostic and treatment options that are almost as advanced as those in human medicine [1]. In that context, Springer and colleagues [1] found that veterinarians’ use of new technologies and other developments is linked to a desire for self-improvement, and is an important source of motivation in their daily work life: Veterinarians may increasingly feel a desire, or an obligation, to advance veterinary medicine in order to provide best and up-to-date patient care [1, 14–16]. Perhaps as a consequence of this, the role of health insurance for dogs and cats is becoming increasingly important as a means to reduce the potential impact of clients’ financial limitations on treatment options [9, 16, 17].

In addition to respecting client’s financial resources, veterinarians also have to understand the client’s emotional bond with the animal. Strong emotional bonds between the client and the animal can have a positive impact on clients’ willingness or motivation to accept costly or prolonged veterinary care [2–4, 7]. However, strong emotional ties between the client and the animal can also have downsides, causing some clients to insist on pursuing advanced and expensive investigations and treatment despite a poor prognosis for the animal [1, 5, 6]. In such situations, the challenge for veterinarians is to consider the patient’s best interests, while showing empathy towards the client’s wishes and emotional needs.

Undoubtedly, the aforementioned challenges in balancing the clients’ emotional needs [1, 5, 6] and financial resources [8–11] with the desire to practice high quality veterinary medicine can result in situations in which veterinarians are torn between patients’, clients’ and their own interests. Further influencing veterinarian’s decision-making processes is the desire to have work-life balance while earning a good living, as illustrated in a UK study, in which 85% of 2,865 veterinary students and graduates highlighted these as key goals for the profession for 2030 [18].

Thus, veterinarians need to manage a number of potentially conflicting expectations and challenges: First, to look after the interests of the patient. Second, to consider clients’ emotional needs, wishes and interests during patient care. Third, to be a competent and skilled veterinarian using up-to-date diagnostic and treatment options. Fourth, to have a fulfilling yet balanced work life. Against this background, we hypothesize that during a consultation, veterinarians draw on four decision-making strategies, which we will refer to as ‘decision ethics orientations’ (DEOs), to balance potentially conflicting issues.

The first DEO, patient-focused decision ethics, is defined as giving priority to the patients’ well-being, by having a primary focus on the best interest of the patient during decision-making processes. The second DEO is referred to as client-empathetic orientation, where there is a greater focus on recognizing and being empathetic to the clients’ emotional needs, wishes, and personal circumstances. The third DEO is referred to as the client-devolved orientation, in which veterinarians prioritize a desire to have a fulfilling work life, by distancing themselves to an extent from the client’s personal needs or problems (e.g. financial limitations or emotional needs). Thus, the veterinarians focus on their responsibility to inform the client about the available diagnostic and treatment options, but ultimately leave the final decision to the client. Lastly, veterinarians may feel a strong responsibility to provide optimal patient care and contribute to the advancement of veterinary medicine [1, 15, 16] leading them to have a more development-oriented DEO. The idea that veterinarians use different strategies to manage complex situations emerging from the three-sided relationship between the patient, the client and the veterinarian was also emphasized by other authors [19, 20]. However, previous studies exploring decision-making strategies present empirical data either from farm animal veterinarians only [20] or from veterinarians working in various fields within the profession [19]. We aspire in our study to conceptualize a framework that meets specific requirements and expectations relating to modern small animal practice. We do not aim to formulate only one DEO for each stakeholder e.g., one DEO for the patient, one DEO for the client and one DEO for the veterinarian, but rather to take into account of a variety of relevant aspects that may impact clinical decision-making. These strategies need not be conscious but could also be culturally formed and utilized without much reflection.

Hence, the present study goes beyond existing research and advances the debate in the following two respects: first, it aims to empirically identify DEOs that are hypothesized to exist among veterinarians working in small animal practice; and secondly, it aims to study their prevalence, socio-demographic and practice-specific origins, and impact on client communication across three different countries.

The methodological approach falls within the field of empirically informed veterinary ethics by describing how veterinarians actually manage expectations and concerns emerging in modern small animal practice. In contrast to the normatively determined approach, where authors, for instance, make a normative claim to the effect that veterinarians *should* focus on patient-centered factors in decision-making processes [21–25], the current study does not aim to promote a certain view on how veterinarians should act. Rather, the four hypothesized DEOs allowed us to identify, present and explain how veterinary decision-making *is actually* shaped. It is important to emphasize that we are not suggesting that some veterinarians predominantly focus only on one type of concern as reflected in a specific DEO and do not consider any others. Instead, we assume that it is more a matter of how individuals balance the four concerns, all of which are relevant to varying extents in most cases.

Specific external factors may influence veterinarians’ decision-making, for example, the existence of pet health insurance [9, 11, 17], which differs in uptake across Europe. For instance, the number of insured dogs varies from well below 10% in Austria [26] up to 90% in Sweden [27]. It could be assumed that different rates of health insurance for dogs and cats will influence DEOs as a higher number of insured animals might reduce the challenge of financial limitations on treatment options and subsequent decision-making.

Another factor that differs across Europe is the number of corporate practices and clinics [28], which has been increasing in recent years [29]. Corporatization of the profession is most extensive in the UK, having started in 1999 [30]. Differences in practice and clinic management, economic aspects, and veterinarians’ autonomy likely impact on professionals’ daily work life [29, 31, 32]. If, for example, standard treatment protocols are in place, veterinarians may have less autonomy during clinical decision-making [32]. Similarly, corporate business models may encourage veterinarians to focus more on business aspects, and create more pressure to earn revenue [32], which might make them less client-empathetic or client-devolved.

Thus, we assume that the uptake of the hypothesized DEOs may not only be determined by the different prevailing interests of the stakeholders in specific situations, but also by the prevalence of health insurance, and different organizational structures of the individual veterinary practice e.g., business type. The aforementioned situations where veterinarians have to handle conflicting expectations and interests relating to the patient, the client and the veterinarian personally have been identified in several countries such as the US [3, 9, 11], the UK [10, 12, 31, 33–35], Austria [1], Denmark [4, 8, 36] and the Netherlands [2, 20]. For this reason, this study includes populations from three countries, to determine whether the hypothesized DEOs are empirically identifiable across three European countries with differing organizational structures around veterinary practices.

Consequently, the aims of the comparative transnational survey across Austria, Denmark and the UK were to investigate (i) whether the four DEOs can be empirically identified among Austrian, Danish, and UK veterinarians, and (ii) to examine whether the prevalence of the different DEOs varies between countries. Further, it was aimed to identify (iii) whether specific factors within countries, socio-demographic or practice-specific, explain any differences in the DEOs. The final aim was to (iv) examine if the uptake of specific DEOs correlates with how frequently veterinarians discuss health insurance for dogs and cats with clients who do not have insurance.

## Materials and methods

### Study population and recruitment of participants

The target group of the transnational study were veterinarians who only or primarily work in the field of small animal practice. To try to ensure comparable study samples across all three countries, participants were recruited in cooperation with small animal veterinary associations in Austria (VÖK–Vereinigung Österreichischer Kleintiermediziner), Denmark (DVA–Danish Veterinary Association, Companion Animal Section) and UK (BSAVA–British Small Animal Veterinary Association). A link to the online questionnaire was sent via e-mail from the respective associations to all their members: the VÖK in Austria (1,195 members), the companion animal group of DVA in Denmark (1,287 members) and BSAVA in UK (5,138 members). Members of the Austrian and Danish associations were invited to complete the online survey between 2^nd^ March and 9^th^ April 2020. The survey for BSAVA members was open from 30^th^ March until 7^th^ May 2020. The invitation provided information about the background of the study, the participating universities, the project funding, ethical approval and participant’s rights during the reply process. Reminder e-mails were sent two weeks after opening the survey.

Since not all small animal veterinarians are members of the relevant veterinary associations in the three countries, a coverage error exists between members of veterinary associations and the ‘true’ number of small animal veterinarians in the three countries. Based on comparison with statistics on the study population, the assumed coverage error is 30% (n = 495) in Austria, and 35.5% (n = 2812) in the UK. For Denmark, no specific data were available on the ‘true’ number of small animal veterinarians. However, the percentage of small animal veterinarians who are members of the DVA is estimated to be around 90%, therefore the assumed coverage error is likely around 10% for Denmark.

### Participants and representativity of the sample

A total of 829 veterinarians clicked on the survey weblink, and 773 (93.2%) went on to answer at least one question. Of the 773 responses, 125 questionnaires were excluded due to a large amount of missing data (e.g., drop-out in section A, section B.1 and B.2 –see S1 Appendix). With a dropout rate of 15% (125/829), the final sample of the current study comprised 648 veterinarians, made up of 102 Austrian veterinarians (15.8%), 172 Danish veterinarians (26.5%) and 374 veterinarians from the UK (57.7%). The response rate was 8.6% for Austria, 13.4% for Denmark and 7.3% for the UK. For the Austrian and UK samples non-response analyses were conducted to ascertain whether the samples deviated from the population census of small animal veterinarians in the two countries. This could not be checked for the Danish data, as relevant census data was not available.

For Austria, it was possible to compare the geographical distribution of the sample data with population census data. A relatively modest deviation was found (see Table 1), which was statistically insignificant at the 0.05 level (Chi-square 2.56(2); p = 0.279). For the UK, it was possible to compare gender and age of the sample data with population census data (Table 2). The proportion of female veterinarians was relatively similar to the study population (only 7% difference), although the difference is statistically significant (Chi-square 7.44(1); p<0.01). There were also statistically significant differences in age groups (Chi-square 58.42(4); p<0.001), with an overrepresentation of young veterinarians (≤25–30 years) and an underrepresentation of veterinarians aged 31 to 40 years in the study sample compared to the study population.

**Table 1:** Analysis of non-response regarding location for Austrian veterinarians (counts (percentage)).

Region	Study population	Study sample
West Austria	499 (26)	33 (33)
East Austria	1.079 (55)	50 (50)
South Austria	377 (19)	17 (17)
**Total**	1.955 (100)	100* (100)

*missing values: 3 (respondents of the study sample who did not indicate their location of work).

**Table 2:** Analysis of non-response regarding age groups and gender in UK (counts (percentage)).

Gender	Study population	Study sample
Males	2.976 (37.4)	113 (30.5)
Females	4.983 (62.6)	257 (69.5)
**Total**	7.959 (100)	370* (100)
**Age group**		
≤25–30	1075 (13.5)	77 (21.7)
31–40	2803 (35.2)	78 (22.0)
41–50	2130 (26.8)	77 (21.7)
51–60	1419 (17.8)	81 (22.8)
≥ 60	527 (6.6)	42 (11.8)
**Total**	7954 (100)	355** (100)

*missing values: 4 (respondents of the study sample who did not indicate their gender).

**missing values: 19 (respondents of the study sample who did not indicate their age).

The discrepancies between census and sample populations raises the question of whether the data should be weighted to take into account over- and underrepresentation of the socio-demographic segments. However, it was decided not to weight the data for the following two reasons: the discrepancy between population census and the UK and Austria samples is modest. Further, as is reported in the result section and detailed in (see S1 and S2 Tables), age and gender (in the UK) had a very limited effect on the investigated DEOs.

### Survey development

The questionnaire content was developed based on results of an Austrian focus group study on modern small animal practice [1]. In addition, a literature review of mainly empirical investigations was conducted to consider further aspects related to the hypothesized DEOs and practice-specific factors prevailing in Danish and UK small animal veterinary practice [3, 4, 8, 9, 11, 12, 31, 33–37]. The questionnaire was developed in English, and a two-step back-translation procedure then used to produce the Austrian and Danish versions. In a first step, the English questionnaire was translated into German and Danish. In a second step, the translated German and Danish versions were translated back into English by a second bilingual person. Subsequently, the retranslations were compared with the original English version. In case of mismatches, alterations were made in the Danish and Austrian version in consultation with translators.

The questionnaire underwent two stages of pre-testing. In the first stage, cognitive interviews [38, 39] were conducted with four Austrian veterinarians with different working backgrounds to identify whether the content led to uncertainties for, or misunderstandings by, the respondent. In a second stage, an online pre-test phase was conducted with 25 small animal veterinarians: ten Austrian, nine Danish and six from the UK. All relevant comments that were likely to improve the quality of data were considered and incorporated into the final version of the questionnaire in all three languages.

The entire project was approved by The Research Ethics Committee of SCIENCE and HEALTH (ReF: 504-0114/19-5000) at the University of Copenhagen and supported by the relevant veterinary associations in the three countries. We ensured informed consent in the following way: before participants were directed to the survey, they were informed that completion of the questionnaire was voluntary, that they could exit the survey at any point, that responses are anonymous and that no personal information (e-mail address etc.) can be traced back to them. By clicking the “next” button, the survey was started. At the end of the survey, participants were able to provide their e-mail address via a separate link if they wished to receive a short summary of survey results.

### Survey design and measurements

The questionnaire consisted of three sections (see S1 Appendix) and was designed so that questions could be skipped, enabling respondents to answer a section even if not all questions were completed in a previous section. For the following detailed description of sections, only those questions and items of relevance to the present paper are considered.

The first section, A, included 14 closed-ended socio-demographic and practice-related questions about age, gender, years of work experience (henceforth referred to as work experience), employment status, business type and geographic location. Questions were also asked about involvement in management, whether individual income depended on practice or clinic income, ability to discount fees and whether post-graduate specialist qualifications were held (e.g. certificate or Board certification).

The second section, B, covered emerging topics in advanced small animal practice, of which 23 statements are related to the hypothesized DEOs that are the focus of the present paper. All 23 statements were formulated to relate to one of the four DEOs. These include statements related to advancements in small animal practice, and an item matrix with statements on factors related to the patient, the client and the veterinarians’ professional environment. The statements were randomly presented to avoid question order bias. Respondents could indicate their level of agreement with each statement through one of seven response options: 1 “strongly disagree”, 2 “disagree”, 3 “somewhat disagree”, 4 “neutral (neither agree nor disagree)”, 5 “somewhat agree”, 6 “agree” and 7 “strongly agree”. Veterinarians were also asked about health insurance for dogs and cats, including how often they discuss it with owners who do not have it. Response options were “Never”, “Occasionally”, “Frequently”, “Always” and “I don’t know”.

Further, two case vignettes were presented in a third section, C. However, data from section C are not relevant to the research questions considered in this paper and so are not presented here.

## Data collection and analysis

All three online surveys were designed and set-up using the survey software Alchemer^®^ (former SurveyGizmo^®^). Data from the Austrian, Danish and UK populations were separately collected via the survey software and merged into one data file.

A large number of tests of association were conducted in this study. Specifically, we performed 36 independent t-tests, 36 one-way ANOVAs, eight multivariate regression analyses including six parameters estimated, and six ordinal regression analyses including eight parameters estimated. Accordingly, adjustment for multiple comparisons in all bivariate tests of association was carried out. The details of these corrections are reported below.

Univariate descriptive statistics were presented in tables, figures or text. Bivariate statistics were conducted for data exploration. For hypotheses testing and research question investigation regression analyses were conducted correcting p-values due to multiple testing. With this correction a maximum of 5% type 1 error rate was ensured.

For bivariate statistics, Chi-square tests (for seven categorical variables) and Kruskal-Wallis *H* tests (for two ordinal variables) were conducted to identify differences between Austria, Denmark and UK with respect to socio-demographic and practice-specific aspects, and are presented in S1 Table. Six of the conducted Chi-square tests show significant differences. In case of significant variables, further Chi-square tests were conducted in order to detect differences between the three sub-populations (see S1 Table). Here, effect size Phi or Cohen’s d were calculated and Bonferroni correction was applied for these multiple comparisons between countries. Hence, only adjusted significant p-values are presented in S1 Table and in the results section.

Aiming to identify the four hypothesized DEOs, principal component analysis (PCA) in combination with an item exclusion strategy was used, and followed up with confirmatory factor analysis. PCA is a method used to identify latent patterns in a set of variables [40]. The 23 statements (hereafter called ‘items’) from section B of the survey were inserted into a PCA, and output with four components (corresponding to the four hypothesized DEOs) was requested. The item exclusion strategy consisted of removal of all items that did not correlate with one of the four DEOs (i.e. correlation coefficient <0.5), or did not correlate with the DEO component they were expected to belong to, or that correlated with several components. As a result, eleven items were removed, leaving twelve remaining items that correlated clearly and strongly with one component while showing only a small or no correlation with the other components. To address whether it was appropriate to carry out latent variable analysis on these twelve items, the Kaiser-Meyer-Olkin measure of sampling adequacy and Bartlett’s test of sphericity is reported. Following this procedure, confirmatory factor analysis (CFA) was run on the final twelve items to assess whether the factorial structure was acceptable, and to assess correlations between the latent variables. To assess the model fit of the DEOs Chi-square values and degree of freedom (df), the standardized root mean square residual (SRMR), root mean square error of approximation (RMSEA), the comparative fit index (CFI), and the Tucker-Lewis index (TLI) are reported [41, 42]. A SRMR and RMSEA value below 0.08, and CFI/TLI values above 0.90 indicate acceptable fit.

Results from this CFA are reported in the form of factor loadings between the items and the designated factors along with correlation coefficients between the four DEOs.

The reliability of the four resulting latent variables were also examined using the Ordinal alpha coefficient measure of internal consistency [43]. The threshold for an acceptable level of internal consistency at alpha 0.6 was set, as suggested for early stages of research [44].

In the end, four latent variables corresponding to the four DEOs identified through the PCA were constructed on the basis of summation of raw scores. This procedure translates the ordinal responses into four variables, one for each DEO, that have interval properties and that can be used in subsequent analyses. The prevalence of the four DEOs using paired sample t-tests (with Bonferroni corrected p-values) was compared. In total, six paired sample t-test were conducted. In order to identify whether socio-demographic and practice-specific factors within each country have an effect on DEOs, independent t-tests (gender, employment type, business type) or one-way ANOVAs (age, work experience, level of qualification) were conducted. Welch corrected values were reported for comparisons with non-homogenous variances. Where significant differences were observed, the loci of these were determined via post hoc analyses using a Games-Howell procedure. Variables effect size were presented using Cohen’s d and Bonferroni correction was applied for these multiple comparisons (see S2 Table).

Data from all countries were then pooled, and multivariate linear regression analyses were run to examine whether work experience or age, employment status, business type and country of work were associated with veterinarians’ DEOs. This was to check whether the socio-demographic factors affected the DEOs irrespective of country, and whether country differences remained after controlling for socio-demographic factors. In total, eight regressions were run. DEO was inserted as outcome (dependent) variable. Variance inflation factor statistics suggested not to include age and working experience as continuous variables in the same models. In order to avoid the problem of multicollinearity, four multivariate regression analyses were conducted initially with working experience (see Table 5) and then with age (see Table 6). Both variables are arguably of importance, since veterinarians’ age might influence their experience and relationship with clients as well as familiarity with and use of technology, and consequently might influence their decisions. The variable work experience seems to be of relevance, because length of training (undergraduate, postgraduate) can vary between countries and individuals, hence veterinarians may start work at different ages. Consequently, veterinarians at the same age can have very different levels of work experience. Based on these reasons, work experience (see Table 5) or age (see Table 6), gender, employment status, business type and country of work were implemented as predictors (independent variables). Additionally, two different sets of regressions were run with different reference categories: first with Austria as the reference category, and then with the UK as the reference category (see Tables 5 and 6). Due to a low number of respondents working at a “University hospital” and “Shelter and/or charity (as an employee)”, and a low number of “Retired (but still professionally active)” veterinarians, we decided to exclude these observations from the analysis.

In total, six ordinal regression analyses were run, one for each country, including first, work experience (see Table 7) and second, age (see Table 8) to identify the extent to which socio-demographic and practice-specific factors as well as DEOs can predict the frequency of discussions about pet health insurance with clients that do not already have it. Frequency of discussions was used as outcome (dependent) variable. The answer “I don’t know” was excluded from this analysis. Gender, employment status and business type were implemented as dichotomous predictor (independent) variables. Work experience (see Table 7) or age (see Table 8) and the four DEOs were included as continuous predictor variables. Again, respondents working at a “University hospital” and “Shelter and/or charity (as an employee)”, and “Retired (but still professionally active)” veterinarians were excluded from this analysis. Spearman’s rho rank correlation coefficients were calculated to identify whether the four DEOs were associated with the frequency of vets discussing pet health insurance with clients that do not already have it. In case of significant associations between DEOs and frequency of discussion, box plots are presented to illustrate how the level of reliance on a specific DEO is correlated to frequency of discussions about health insurance.

IBM^®^ SPSS^®^ Statistics version 26 was used for univariate and bivariate statistics. For the confirmatory factor analysis (CFA) where Mplus^®^ version 8.4 was used.

## Results

### Socio-demographic and practice-specific factors

Overall, 181 (28.1%) male and 462 (71.9%) female veterinarians participated in the study. The mean age of the study population was 44.7±12.5 years. The mean age of male respondents was 50.2±13.2 years; and of their female colleagues was 42.6±11.7 years. Detailed information on socio-demographic and practice-specific factors for the whole study population, and for each sub-population from Austria, Denmark and UK, is listed in S1 Table.

There were clear and significant differences between the countries in practice-specific factors such as business type, employment status, involvement in the daily management, dependency of veterinarians’ income on the income of the practice or clinic and permission to discount client fees. More veterinarians in the UK worked in corporate owned practices or clinics compared to veterinarians from Austria (χ^2^(1) = 91.324, p<0.001) and Denmark (χ^2^(1) = 65.459, p<0.001). Further, veterinarians in the UK were less able to discount fees without permission compared to Austrian (χ^2^(1) = 40.589, p<0.001) and Danish (χ^2^(1) = 46.179, p<0.001) colleagues. In contrast, in Austria more veterinarians were self-employed compared to in Denmark (χ^2^(1) = 46.230, p<0.001) and UK (χ^2^(1) = 108.329, p<0.001) or worked in independently owned practices or clinics (Denmark: χ^2^(1) = 14.430, p<0.001; UK: χ^2^(1) = 91.324, p<0.001). Further, Austrian veterinarians were more involved in the daily management compared to their UK (χ^2^(1) = 30.207, p<0.001) and Danish (χ^2^(1) = 38.413, p<0.001) colleagues. Compared to veterinarians in Austria (χ^2^(1) = 28.134, p<0.001) and the UK (χ^2^(1) = 40.097, p<0.001), Danish veterinarians’ income was less dependent on the income of the practice or clinic. Further, a higher proportion of Danish veterinarians had a clinical postgraduate qualification compared to Austrian (χ^2^(2) = 27.726, p<0.001) and UK veterinarians (χ^2^(2) = 31.155, p<0.001). However, due to different options and levels of postgraduate clinical qualifications among sub-populations (e.g. Fachtierarzt in German speaking countries, specific Master programmes in Denmark) the observed differences in postgraduate qualification needs to be carefully interpreted. Details of these analyses are given in S1 Table.

### Identification of four DEOs

Twelve items were retained after the item removal strategy (described in the Data collection and analyses section). Both the Bartlett test of sphericity (χ2(66) = 1144.710, p<0.001) and the Kaiser-Mayer-Olkin measure of sampling adequacy (KMO = 0.66) confirmed that those variables were suitable for PCA. The four-factor solution was supported by the Kaiser criterion of eigen values for the extracted components greater than 1 and the elbow criterion in the scree plot (results not shown). The four factors explained 59.1% of the variances. The individual rotated components resulted in 18.5%, 15.9%, 13.8% and 10.9% of the total variances. Results from subsequent confirmatory factor analysis (CFA) showed an acceptable fit with the data (CFI 0.94; TLI 0.92; RMSEA 0.047; SRMR 0.042). Ordinal alpha reliability coefficients ranged from 0.64–0.77.

In general, the four latent components were found to match our expectations of the hypothesized DEOs presented in the introduction (see Table 3). The three items that loaded on component 1 (meaning that the statements are correlated with each other) related to veterinarians’ empathy with clients’ emotional well-being and personal circumstances during decision-making processes. Therefore, this component was labelled “client-empathetic”. The three items that loaded on the second component related to veterinarians’ attitudes towards the development of small animal practice, was therefore labelled “development-oriented”. The three items that loaded on component 3 related to small animal veterinarians’ focus on patient’s interests during decision-making processes. This component was labelled “patient-focused”. The last three items loaded on component 4 and concerned veterinarians’ tendency to delegate the decision-making to the client, which was therefore labelled “client-devolved”. In the bottom part of Table 3, correlations between the DEOs are displayed. While some of the orientations are significantly associated with each other, the correlations are relatively modest, and in all cases smaller than 0.3.

**Table 3:** Results from confirmatory factor analysis for the identification of veterinarians’ Decision Ethics Orientations (DEOs) (N = 623).

	Component 1:	Component 2:	Component 3:	Component 4:
	Client- empathetic	Development- oriented	Patient- focused	Client-devolved
It is important to act not only professionally, but also to be emotionally supportive to the client.	0.759			
I empathise with lonely and elderly clients in my decision-making processes.	0.601			
It is important to empathise with the client’s emotions in the decision-making process.	0.627			
Veterinary medicine should offer the same diagnostic options as human medicine.		0.441		
It is important to promote the advancement of small animal medicine for future patients.		0.634		
It is important for the veterinary profession to keep developing innovative methods, even though it is impossible to predict possible complications.		0.576		
It is more important for me to act in the best interest of the patient than of the client.			0.670	
The patient is my first priority when I make medical decisions.			0.580	
It is important to make decisions that are in the patient’s best interests, even if I might lose the owner as client.			0.478	
I do not make the decisions—the client has to make them for the patient.				0.789
In situations where my opinion differs from that of the client, I will ultimately let the client decide.				0.627
I advise the client about all possible treatment options, but it is up to the client to decide between them.				0.546
***Ordinal alpha reliability coefficients***	*0*.*77*	*0*.*64*	*0*.*65*	*0*.*71*
**Correlations between DEOs**				
Client-empathetic	1.000			
Development-oriented	0.238**	1.000		
Patient-focused	0.125*	0.295**	1.000	
Client-devolved	0.092	-0.074	0.041	1.000

Results from Confirmatory Factor Analysis with standardized factor scores and correlations (using the stdyx method). CFA model fit results: X^2^ 114.3(48); CFI 0.94; TLI 0.92 RMSEA 0.047; SRMR 0.042.

*correlation is significant at the 0.05 level (2-tailed).

**correlation is significant at the 0.01 level (2-tailed).

Table 4 presents the level of agreement for all items for the DEOs. A value of 4 indicates a neutral attitude towards the statements, with less than 4 indicating disagreement, and above 4 indicating agreement. In all countries, we observe quite substantial differences between veterinarians (i.e. many agree and many disagree) in response to the development-oriented statements (“It is important for the veterinary profession to keep developing innovative methods, even though it is impossible to predict possible complications” and “Veterinary medicine should offer the same diagnostic options as human medicine”). A similar tendency is observed for the client-devolved statements (“I do not make the decisions—the client has to make them for the patient” and “In situations where my opinion differs from that of the client, I will ultimately let the client decide”) except for the statement “I advise the client about all possible treatment options, but it is up to the client to decide between them” where a large majority agrees. Across countries, veterinarians have a tendency to agree with the patient-focused and client-empathetic statements, although a small proportion of veterinarians were neutral or disagreed in the item statements. We assessed whether the patterns observed at the single-item level were also statistically significant at the latent variable level. To do this the four variables measuring the four DEOs (described in the method section) were constructed, and we subsequently ran six paired sample t-tests with Bonferroni corrections (means are reported in Table 4). The client-empathetic orientation was found to be more prevalent than the development-oriented (t(647) = 22.502, p<0.001), the client-devolved (t(647) = 22.306, p<0.001), and the patient-focused (t(647) = 11.829, p<0.001) orientations. Further, the patient-focused orientation is more prevalent than the development-oriented (t(674) = 11.889, p<0.001) or the client-devolved (t(647) = 11.112, p<0.001) orientations. Comparison between client-devolved and development-oriented DEO’s did not identify a significant difference (t(674) = 0.490, p = 0.625).

**Table 4:** Level of agreement on 12 items related to four Decision Ethics Orientations (DEOs).

		All countries		Austria		Denmark		UK	
		(N = 642–648)		(n = 101–102)		(n = 170–172)		(n = 370–374)	
		n (%)	M±SD	n (%)	M±SD	n (%)	M±SD	n (%)	M±SD
It is important to act not only professionally, but also to be emotionally supportive to the client.	1–3 Disagreement	14 (2.2)	6.0±1.0	6 (5.9)	5.7±1.2	1 (0.4)	6.2±0.8	7 (1.9)	6.0±1.0
	4	25 (3.9)		7 (6.9)		4 (2.3)		14 (3.8)	
	Neutral								
	5–7 Agreement	606 (93.9)		89 (87.2)		167 (97.3)		350 (94.3)	
I empathise with lonely and elderly clients in my decision-making processes.	1–3 Disagreement	26 (4.0)	5.9±1.1	8 (7.9)	5.5±1.3	8 (4.7)	5.7±1.1	10 (2.7)	6.0±1.0
	4	45 (7.0)		12 (11.9)		18 (10.5)		15 (4.0)	
	Neutral								
	5–7 Agreement	575 (89.0)		81 (80.2)		146 (84.8)		348 (93.3)	
It is important to empathise with the client’s emotions in the decision-making process.	1–3 Disagreement	26 (4.0)	6.1±1.1	13 (12.9)	5.2±1.4	1 (0.6)	6.5±0.7	12 (3.2)	6.1±1.0
	4	17 (2.6)		8 (7.9)		1 (0.6)		8 (2.2)	
	Neutral								
	5–7 Agreement	599 (93.4)		119 (79.2)		168 (89.5)		352 (94.6)	
**Client-empathetic**			**6.0 ±0.8**		**5.5 ±1.1**		**6.1 ±0.7**		**6.0 ±0.8**
Veterinary medicine should offer the same diagnostic options as human medicine.	1–3 Disagreement	226 (34.9)	4.5±1.6	15 (14.7)	5.1±1.5	45 (26.2)	4.7±1.6	165 (44.4)	4.2±1.5
	4	57 (8.8)		6 (5.9)		13 (7.6)		38 (10.2)	
	Neutral								
	5–7 Agreement	365 (56.3)		81 (79.4)		114 (66.3)		170 (45.4)	
It is important to promote the advancement of small animal medicine for future patients.	1–3 Disagreement	106 (16.4)	5.4±1.3	4 (4.0)	5.7±1.2	3 (1.8)	6.0±1.0	99 (26.5)	5.1±1.4
	4	33 (5.1)		11 (10.9)		9 (5.2)		13 (3.5)	
	Neutral								
	5–7 Agreement	508 (78.5)		86 (85.1)		160 (93.0)		262 (70.0)	
It is important for the veterinary profession to keep developing innovative methods, even though it is impossible to predict possible complications.	1–3 Disagreement	191 (29.5)	4.7±1.4	19 (18.6)	4.7±1.4	10 (8.7)	5.2±1.3	157 (42.0)	4.4±1.4
	4	96 (14.8)		25 (24.5)		31 (18.1)		40 (10.7)	
	Neutral								
	5–7 Agreement	360 (55.7)		58 (56.9)		125 (73.0)		177 (47.3)	
**Development-oriented**			**4.9 ±1.0**		**5.2 ±1.0**		**5.3 ±1.0**		**4.6 ± 1.0**
It is more important for me to act in the best interest of the patient than of the client.	1–3 Disagreement	62 (9.7)	5.3±1.2	3 (1.8)	5.3±1.1	17 (10.0)	5.3±1.3	42 (11.4)	5.2±1.2
	4	87 (13.6)		15 (14.9)		28 (16.4)		44 (11.9)	
	Neutral								
	5–7 Agreement	493 (76.7)		83 (82.3)		126 (73.6)		284 (76.8)	
The patient is my first priority when I make medical decisions.	1–3 Disagreement	29 (4.5)	5.8±1.2	2 (2.0)	6.0±0.9	7 (4.1)	5.8±1.1	20 (5.4)	5.8±1.1
	4	36 (5.6)		-		15 (8.8)		21 (5.7)	
	Neutral								
	5–7 Agreement	579 (89.9)		100 (89.0)		149 (87.1)		330 (88.9)	
It is important to make decisions that are in the patient’s best interests, even if I might lose the owner as client.	1–3 Disagreement	64 (9.9)	5.2±1.2	6 (5.9)	5.2±1.1	16 (9.3)	5.3±1.2	42 (11.4)	5.2±1.2
	4	84 (13.0)		17 (16.7)		22 (12.8)		45 (12.1)	
	Neutral								
	5–7 Agreement	491 (76.1)		79 (77.4)		134 (77.9)		284 (76.5)	
**Patient-focused**			**5.4 ±0.9**		**5.5 ±0.8**		**5.5 ±0.9**		**5.4 ±0.9**
I do not make the decisions—the client has to make them for the patient.	1–3 Disagreement	258 (39.9)	4.1±1.6	47 (46.1)	3.6±1.5	76 (44.1)	4.1±1.6	135 (36.2)	4.2±1.5
	4	87 (13.4)		20 (19.6)		16 (9.3)		51 (13.7)	
	Neutral								
	5–7 Agreement	302 (46.7)		35 (34.3)		80 (46.6)		187 (50.1)	
In situations where my opinion differs from that of the client, I will ultimately let the client decide.	1–3 Disagreement	150 (23.2)	4.7±1.3	32 (31.4)	4.3±1.4	44 (26.2)	4.6±1.4	73 (19.6)	4.8±1.3
	4	107 (16.6)		19 (18.6)		26 (15.1)		62 (16.7)	
	Neutral								
	5–7 Agreement	389 (60.2)		51 (50.0)		101 (58.7)		237 (63.7)	
I advise the client about all possible treatment options, but it is up to the client to decide between them.	1–3 Disagreement	44 (6.8)	5.8±1.2	14 (13.8)	5.4±1.4	3 (1.8)	6.0±1.0	27 (7.2)	5.8±1.2
	4	19 (2.9)		6 (5.9)		9 (5.3)		4 (1.1)	
	Neutral								
	5–7 Agreement	583 (90.3)		82 (80.3)		159 (92.9)		342 (91.7)	
**Client-devolved**			**4.8 ±1.071**		**4.4 ±1.2**		**4.9 ±1.0**		**4.9 ±1.0**

Answer option from 7-point-Likert-scale were collapsed: Disagreement = 1 “strongly disagree”, 2 “disagree”and 3 “somewhat disagree”, Neutral = 4 neutral (neither agree nor disagree), Agreement = 5 “somewhat agree”, 6 “agree” and 7 “strongly agree”.

### Effects of socio-demographic and practice-specific factors on the DEOs within each country

By means of country-specific analysis, we aimed to identify to what extent socio-demographic and practice-specific factors have an effect on the reliance on each of the four DEOs within each country. Results suggest that female respondents were less development-oriented than their male colleagues in the UK (t(368) = 3.210, p = 0.001 –see S2 Table). In addition, less experienced veterinarians in the UK were more oriented towards client-devolved DEOs than their colleagues with more than 20 years of experience (F(3,128.80) = 4.451, p = 0.015 –see S2 Table). Furthermore, business type accounted for significant differences for the patient-focused DEO in the UK, with veterinarians working in independently owned practices having a higher score than colleagues working in corporate owned practices (t(332) = 2.349, p = 0.019 –see S2 Table). When considering employment status, employed Austrian veterinarians were significantly more client-devolved oriented compared to self-employed colleagues (t(99) = -2.373, p = 0.020 –see S2 Table).

### Effects of socio-demographic and practice-specific factors on the DEOs across countries

Data from all countries were pooled, and eight multivariate linear regression analyses (one for each of the DEO variables) were run to examine whether work experience or age, employment status, business type and country of work were associated with veterinarians’ DEOs, in part to check whether the socio-demographic factors affected the DEOs irrespective of country, and in part to check whether country differences remain after controlling for socio-demographic factors. Findings indicated that veterinarians’ country of work, work experience and age have a particular influence on the client-empathetic, client-devolved and development-oriented DEOs. As described in the Methods section, to avoid multicollinearity issues, separate regressions are reported where work experience (see Table 5), or age is used as predictor variable (see Table 6).

**Table 5:** Multivariate regression analyses of predictors (including socio-demographic (with work experience), practice-specific factors, and country) of the four Decision Ethics Orientations (DEOs).

**Model 1: Client-empathetic decision ethics orientation**					
Adj. R^2^ = 0.084, F(6,557) = 9.602, p<0.001					
	Unstandardized coefficients		Standardized Coefficient		
	B	Std. Error	Beta	t	Sig.
(Constant)	4.967	.227		21.839	0.000
AT_UK*	.522	.113	.306	4.613	0.000
AT_DK*	.604	.112	.317	5.388	0.000
UK_DK**	.082	.086	.043	.950	0.343
gender	.099	.081	.052	1.224	0.222
work experience	.009	.003	.138	3.016	0.003
business type	-.055	.084	-.031	-.658	0.511
employment type	.167	.089	.096	1.877	0.061
**Model 2: Development-oriented decision ethics orientation**					
Adj. R^2^ = 0.088, F(6, 557) = 10.109, p<0.001					
	Unstandardized coefficients		Standardized Coefficient		
	B	Std. Error	Beta	t	Sig.
(Constant)	5.716	.274		20.849	0.000
AT_UK*	-.458	.136	-.222	-3.352	0.001
AT_DK*	.180	.135	.078	1.333	0.183
UK_DK**	.638	.104	.277	6.115	0.000
gender	-.203	.098	-.088	-2.077	0.038
work experience	-.008	.004	-.102	-2.236	0.026
business type	-.064	.101	-.030	-.628	0.530
employment type	-.019	.107	-.009	-.176	0.860
**Model 3: Patient-focused decision ethics orientation**					
Adj. R^2^ = .003, F(6, 557) = 1.303, p = 0.254					
	Unstandardized coefficients		Standardized Coefficient		
	B	Std. Error	Beta	t	Sig.
(Constant)	5.788	.244		23.749	0.000
AT_UK*	-.006	.121	-.003	-.047	0.962
AT_DK*	-.072	.120	-.037	-.597	0.551
UK_DK**	-.066	.093	-.034	-.712	0.477
gender	-.038	.087	-.019	-.436	0.663
work experience	-.001	.003	-.009	-.189	0.851
business type	-.228	.090	-.126	-2.536	0.011
employment type	.033	.095	.018	.354	0.730
**Model 4: Client-devolved decision ethics orientation**					
Adj. R^2^ = 0.042, F(6, 557) = 5.142, p<0.001					
	Unstandardized coefficients		Standardized Coefficient		
	B	Std. Error	Beta	t	Sig.
(Constant)	4.231	.296		14.316	0.000
AT_UK*	.472	.147	.218	3.209	0.001
AT_DK*	.461	.146	.191	3.165	0.002
UK_DK**	-.011	.112	-.004	-.097	0.932
gender	.044	.105	.081	.418	0.676
work experience	-.007	.004	-.081	-1.733	0.084
business type	.153	.109	.069	1.406	0.160
employment type	.047	.116	.021	.406	0.685

*Austria is reference category.

**UK is reference category.

**Table 6:** Multivariate regression analyses of predictors (including socio-demographic (with age), practice-specific factors, and country) of the four Decision Ethics Orientations (DEOs).

**Model 1: Client-empathetic decision ethics orientation**					
Adj. R^2^ = 0.087, F(6,532) = 8.491, p<0.001					
	Unstandardized coefficients		Standardized Coefficient		
	B	Std. Error	Beta	t	Sig.
(Constant)	4.777	.300		15.906	0.000
AT_UK*	.558	.116	.325	4.792	0.000
AT_DK*	.604	.116	.315	5.222	0.000
UK_DK**	.046	.090	.024	.508	0.612
gender	.074	.084	.038	.882	0.378
age	.009	.003	.123	2.599	0.010
business type	-.061	.087	-.035	-.700	0.484
employment type	.169	.094	.095	1.805	0.072
**Model 2: Development-oriented decision ethics orientation**					
Adj. R^2^ = 0.087, F(6, 532) = 9.142, p<0.001					
	Unstandardized coefficients		Standardized Coefficient		
	B	Std. Error	Beta	t	Sig.
(Constant)	5.716	.274		20.849	0.000
AT_UK*	-.514	.139	-.249	-3.699	0.000
AT_DK*	.121	.138	.053	.877	0.381
UK_DK**	.635	.107	.276	5.912	0.000
gender	-.226	.100	-.097	-2.254	0.025
age	-.010	.004	-.125	-2.660	0.008
business type	-.032	.104	-.015	-.307	0.759
employment type	-.049	.112	-.023	-.441	0.659
**Model 3: Patient-focused decision ethics orientation**					
Adj. R^2^ = .004, F(6, 532) = 1.365, p = 0.227					
	Unstandardized coefficients		Standardized Coefficient		
	B	Std. Error	Beta	t	Sig.
(Constant)	5.850	.317		18.480	0.000
AT_UK*	-.025	.123	-.014	-.202	0.840
AT_DK*	-.121	.122	-.062	-.989	0.323
UK_DK**	-.096	.095	-.049	-1.009	0.313
gender	-.056	.089	-.038	-.630	0.529
age	-.001	.003	-.009	-.179	0.858
business type	-.226	.092	-.126	-2.463	0.014
employment type	.032	.099	.018	.328	0.743
**Model 4: Client-devolved decision ethics orientation**					
Adj. R^2^ = 0.046, F(6, 532) = 5.319, p<0.001					
	Unstandardized coefficients		Standardized Coefficient		
	B	Std. Error	Beta	t	B
(Constant)	4.442	.386		11.511	0.000
AT_UK*	.430	.150	.198	2.873	0.004
AT_DK*	.435	.149	.179	2.926	0.004
UK_DK**	.005	.116	.002	.043	0.966
gender	.039	.108	.016	.358	0.720
age	-.008	.004	-.091	-1.903	0.058
business type	.145	.112	.065	1.301	0.194
employment type	.087	.120	.039	.726	0.486

*Austria is reference category.

**UK is reference category.

Country, veterinarians’ working experience, and age significantly affected the client-empathetic DEO. Older veterinarians (p = 0.010) or more experienced veterinarians (p = 0.003) were more inclined towards the client-empathetic orientation. UK and Danish veterinarians had significantly higher (p<0.001) client-empathetic DEO scores than Austrian veterinarians. In respect to the development-oriented DEO, Austrian (p = 0.001) and Danish (p<0.001) veterinarians were more inclined towards this orientation than UK veterinarians. Further, younger veterinarians (p = 0.008), veterinarians with less experience (p = 0.026) and male veterinarians (p = 0.038 –see Table 5; p = 0.025 –see Table 6) were more development-oriented than their older, more experienced or female colleagues.

Turning to the client-devolved DEO, veterinarians’ country of work had a significant effect. Here, Austrian veterinarians were less inclined towards the client-devolved DEO compared to veterinarians in Denmark (p = 0.002 –see Table 5; p = 0.004 –see Table 6) and the UK (p = 0.001 –see Table 5; p = 0.004 –see Table 6). The patient-focused orientation was not affected by any of the included variables either in the multivariate regression models with age or with working experience (see Tables 5 and 6).

### Frequency of discussing health insurance with owners, and correlation with veterinarian’s DEOs

**Fig 1:**
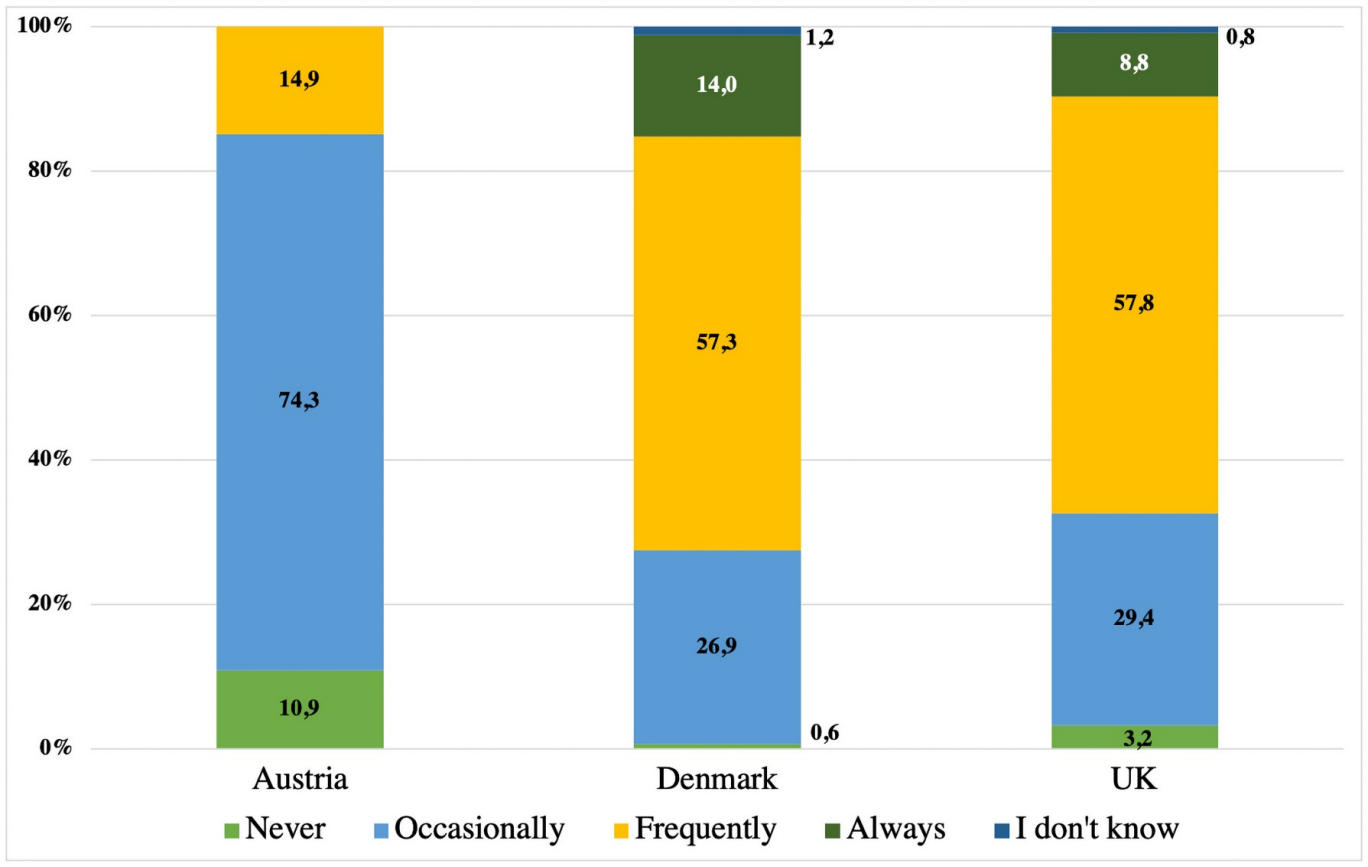
Frequency of discussions among veterinarians and dog and cat owners about the possibility of health insurance in Austria (n = 101), Denmark (n = 171) and the UK (n = 374).

Austrian, Danish and UK veterinarians differed in how often they would discuss health insurance with cat and dog owners (H(2) = 37.111, p<0.001) (see Fig 1). Pairwise comparison indicated that Austrian respondents were significantly less likely (p<0.001) to discuss health insurance with owners of non-insured patients than were their Danish and UK colleagues. Thus, only 14.9% (n = 15) of the Austrian veterinarians reported that they did so frequently. This is in stark contrast to veterinarians in Denmark and UK, where 57.3% (n = 98) and 57.8% (n = 216), respectively, responded that they ‘frequently’ discuss insurance with owners of non-insured patients. Health insurance is ‘always’ addressed by 14.0% (n = 24) Danish and 8.8% (n = 33) UK veterinarians (see Fig 1).

Six ordinal regression analysis were conducted to examine whether the four DEOs can predict the frequency of discussions with clients about health insurance, while controlling for socio-demographic and practice-specific factors. Two separate analyses were conducted with working experience (see Table 7) and age (see Table 8), respectively, inserted. Correlation coefficients were calculated to identify whether the four DEOs were associated with the frequency of discussion about the possibility of health insurance.

**Table 7:** Ordinal regression analysis (including work experience) to predict frequency of discussion among veterinarians and dog and cat owners about health insurance.

**Austria**							
Adj. R^2^ = 0.024; Chi^2^(8) = 1.749, p = 0.988							
						95% confidence interval	
	Estimate	Std. Error	Wald	df	Sig.	Lower Bound	Upper Bound
work experience	-.004	.034	.017	1	0.896	-.072	.063
client-empathetic	.053	.218	.060	1	0.807	-.374	.481
development-oriented	.189	.262	.519	1	0.471	-.325	.703
patient-focused	.008	.321	.001	1	0.980	-6.21	.638
client-devolved	.003	.208	.000	1	0.989	-.405	.411
gender	-.266	.594	.201	1	0.654	-1.430	.898
business type	.058	1.831	.001	1	0.975	-3.530	3.646
employment type	-.312	.807	.150	1	0.699	-1.894	1.269
**Denmark**							
Adj. R^2^ = 0.176; Chi^2^(8) = 24.291, p = 0.002							
						95% confidence interval	
	Estimate	Std. Error	Wald	df	Sig.	Lower Bound	Upper Bound
work experience	.007	.015	.187	1	0.666	-.023	.036
client-empathetic	.173	.264	.432	1	0.511	-.343	.690
development-oriented	.370	.179	4.285	1	0.038	.020	.719
patient-focused	.199	.184	1.162	1	0.281	-.163	.560
client-devolved	-.121	.172	.495	1	0.482	-.458	.216
gender	-.844	.438	3.702	1	0.054	-1.703	.016
business type	-1.908	.503	14.361	1	0.000	-2.895	-.921
employment type	.741	.417	3.160	1	0.075	-.076	1.558
**UK**							
Adj. R^2^ = 0.085; Chi^2^(8) = 23.940, p = 0.002							
						95% confidence interval	
	Estimate	Std. Error	Wald	df	Sig.	Lower Bound	Upper Bound
work experience	-.004	.010	.179	1	0.672	-.023	.015
client-empathetic	.351	.151	4.369	1	0.037	.020	.611
development-oriented	.427	.124	11.903	1	0.001	.184	.669
patient-focused	-.076	.133	.325	1	0.569	-.337	.185
client-devolved	-.116	.111	1.094	1	0.296	-.333	.101
gender	-.365	.264	1.917	1	0.166	-.883	.152
business type	-.349	.243	2.057	1	0.152	-.826	.128
employment type	.076	.300	.065	1	0.799	-.511	.664

**Table 8:** Ordinal regression analysis (including age) to predict frequency of discussion among veterinarians and dog and cat owners about health insurance.

**Austria**							
Adj. R^2^ = 0.028; Chi^2^(8) = 1.949, p = 0.983							
						95% confidence interval	
	Estimate	Std. Error	Wald	df	Sig.	Lower Bound	Upper Bound
age	-.032	.038	.0732	1	0.392	-.107	.042
client-empathetic	.089	.220	.165	1	0.684	-.342	.521
development-oriented	.185	.263	.498	1	0.480	-.329	.700
patient-focused	-.050	.322	.024	1	0.877	-.680	.581
client-devolved	-.024	.211	.013	1	0.909	-.437	.389
gender	.015	.619	.001	1	0.980	-1.198	1.229
business type	-.196	1.857	.011	1	0.916	-3.836	3.444
employment type	-.132	.869	.023	1	0.880	-1.572	1.836
**Denmark**							
Adj. R^2^ = 0.170; Chi^2^(8) = 22.280, p = 0.004							
						95% confidence interval	
	Estimate	Std. Error	Wald	df	Sig.	Lower Bound	Upper Bound
age	.019	.017	1.149	1	0.284	-.016	.053
client-empathetic	.154	.267	.334	1	0.563	-.369	.687
development-oriented	.387	.187	4.270	1	0.039	.020	.753
patient-focused	.248	.194	1.632	1	0.201	-.133	.629
client-devolved	-.107	.176	.371	1	0.543	-.452	.238
gender	-.730	.448	2.655	1	0.103	-1.608	.148
business type	-1.905	.525	13.173	1	0.000	-2.934	-.876
employment type	.555	.437	1.612	1	0.204	-.302	1.412
**UK**							
Adj. R^2^ = 0.082; Chi^2^(8) = 22.112, p = 0.005							
						95% confidence interval	
	Estimate	Std. Error	Wald	df	Sig.	Lower Bound	Upper Bound
age	-.002	.010	.026	1	0.873	-.021	.018
client-empathetic	.358	.153	5.489	1	0.019	.058	.657
development-oriented	.427	.125	11.686	1	0.001	.182	.657
patient-focused	-.076	.136	.312	1	0.576	-.342	.190
client-devolved	-.049	.114	.187	1	0.665	-.272	.173
gender	-.354	.268	1.750	1	0.186	-.878	.170
business type	-.285	.248	1.327	1	0.249	-.769	.200
employment type	.192	.315	.370	1	0.543	-.426	.810

**Fig 2:**
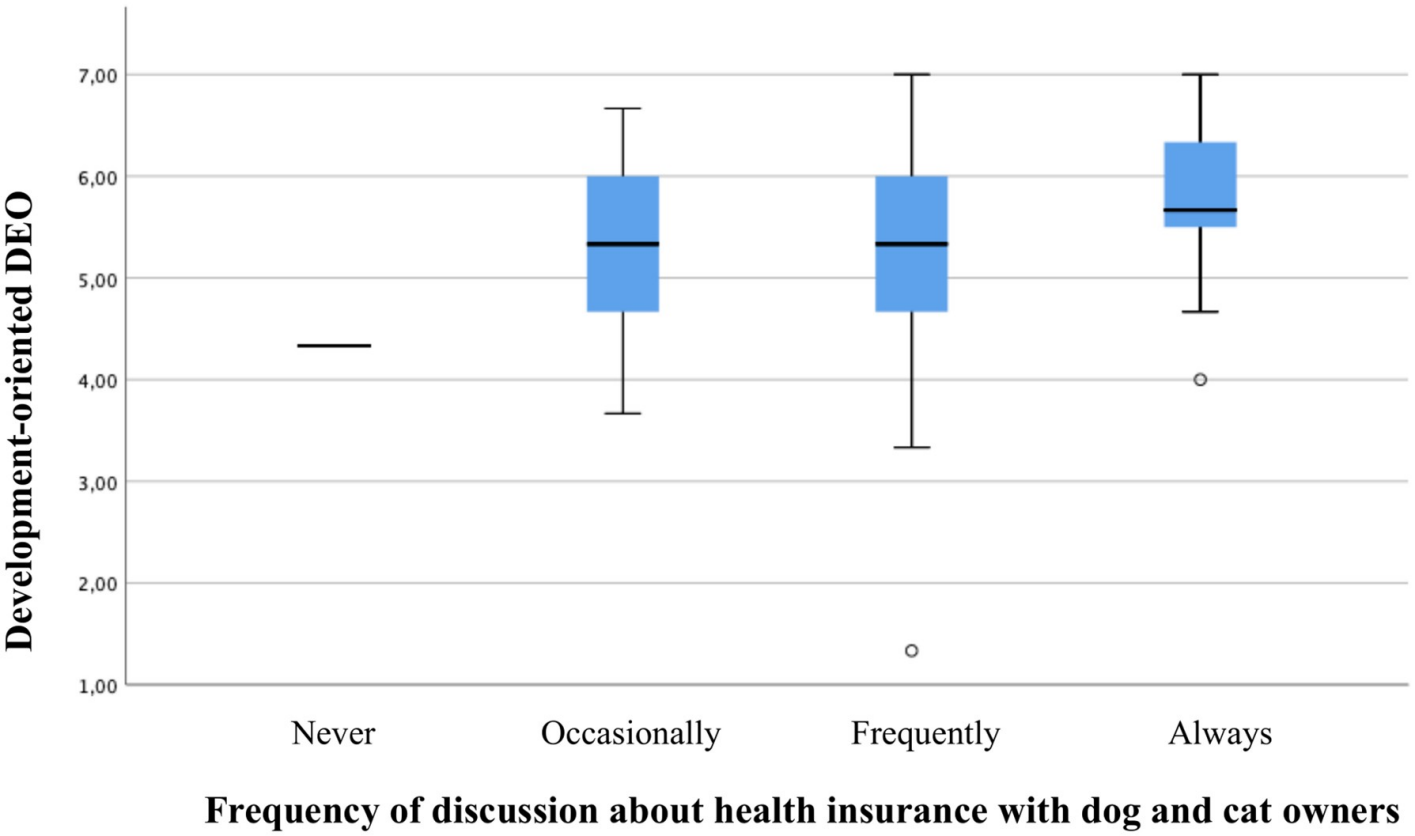
Box plots showing level of development-oriented DEO with respect to frequency of discussion about health insurance for Danish veterinarians. In the box plots, the boundary of the box closest to zero indicates the first quartile, the thick black within the box marks the median, the boundary of the box farthest from zero indicates the third quartile. Whiskers above and below the box indicate 10th and 90th percentiles. Points below the whiskers indicate outliers outside the 10th and 90th percentiles.

**Fig 3:**
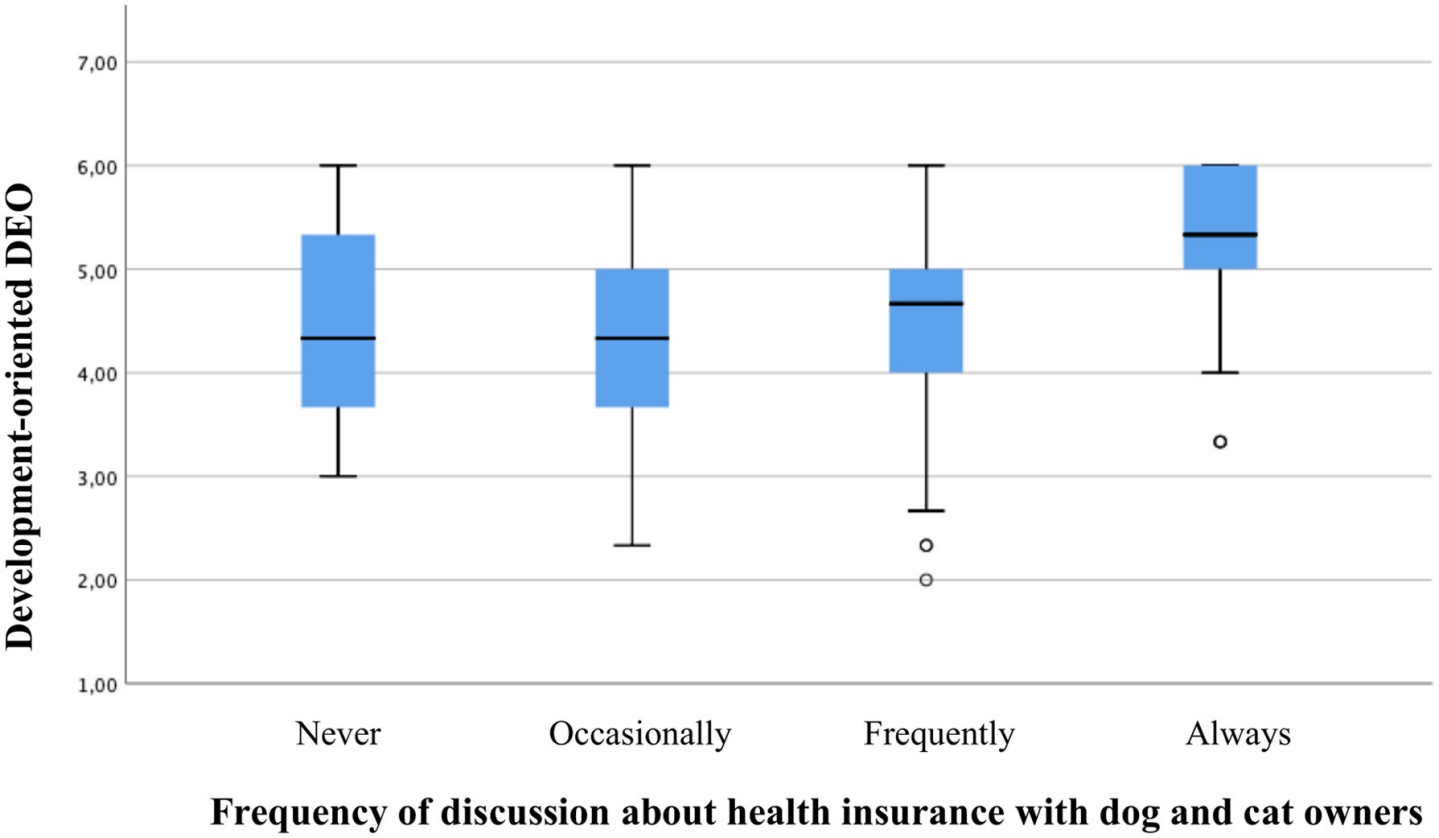
Box plots showing level of development-oriented DEO with respect to frequency of discussion about health insurance for UK veterinarians. In the box plots, the boundary of the box closest to zero indicates the first quartile, the thick black within the box marks the median, the boundary of the box farthest from zero indicates the third quartile. Whiskers above and below the box indicate 10th and 90th percentiles. Points below the whiskers indicate outliers outside the 10th and 90th percentiles.

**Fig 4:**
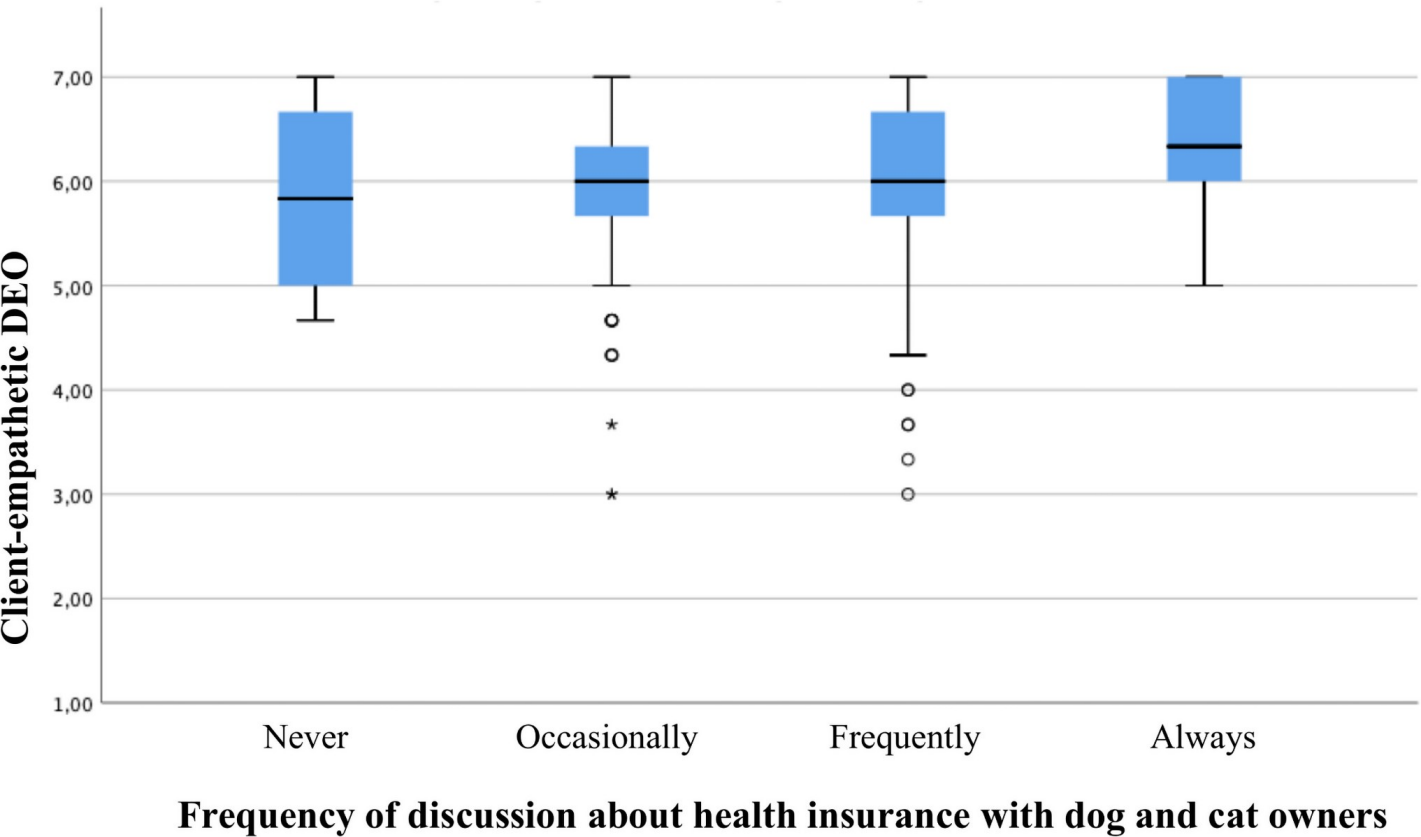
Box plots showing level of client-empathetic DEO with respect to frequency of discussion about health insurance for UK veterinarians. In the box plots, the boundary of the box closest to zero indicates the first quartile, the thick black within the box marks the median, the boundary of the box farthest from zero indicates the third quartile. Whiskers above and below the box indicate 10th and 90th percentiles. Points and asterisk (extreme outliers) below the whiskers indicate outliers outside the 10th and 90th percentiles.

Ordinal regression analysis per country indicated only significant effects for Denmark and the UK. An increase in the development-oriented DEO was correlated with a higher frequency of discussion in Denmark (p = 0.038 –see Table 7; p = 0.039 –see Table 8; r_s_ = 0.189, p = 0.013) and the UK (p = 0.001 –see Table 7; p = .001 –see Table 8; r_s_ = 0.191, p<0.001). In addition, an increase in the client-empathetic DEO was associated with more discussion about health insurance in the UK (p = 0.037 –see Table 7; p = 0.019 –see Table 8; r_s_ = 0.143, p = 0.006). Further, it was found that Danish veterinarians working in independent practices and clinics were less inclined to discuss health insurance during consultations compared to their colleagues working within a corporate business model (p<0.001 –see Table 7; p<0.001 –see Table 8). Figs 2 to 4 presents box plots to illustrate data distribution for significant associations between DEOs and frequency of discussion.

## Discussion

Various empirical studies have focused on factors relating to the patient, the client and the veterinarian that may influence decision-making processes in small animal practice. However to date, limited empirical research has been undertaken to conceptualize possible models by identifying potential strategies to manage competing expectations and concerns in practice. Two relevant empirical studies were identified that provide models of veterinarians’ decision strategies: one with farm animal veterinarians [20], the other with veterinarians working in different veterinary professions [19]. Based on their findings, de Graaf [20] presents four different discourses on animals and owners, and Morgan [19] classified her results along four different models that highlight different prioritization of interests related to the patient and/or the client [19]. Both authors describe veterinarians who prioritize patients’ interests in clinical consultation. Morgan presents this model as “Animal Advocate Model” and de Graaf as “Animal’s Advocates”, which corresponds with our patient-focused DEO. In our present study, the large majority of veterinarians from all three countries agreed with statements related to the patient-focused DEO, indicating the importance of prioritizing patient’s interests over client’s wishes, even at the risk of losing the client.

Further, Morgan introduced the “Information Provider Model”, where veterinarians recognize their professional responsibility to provide information, but believe the client should ultimately be the decision-maker [19]. This reflects our client-devolved DEO, including the statements that veterinarians “advise the client about all possible treatment options, but it is up to the client to decide between them” or veterinarians “do not make the decisions–the client has to make them for the patient”. Comparisons with the present study findings should be made with caution however, as Morgan’s [19] and de Graaf’s [20] results are based on qualitative research designs and did not focus specifically on the small animal sector nor on DEOs explicitly.

Other models have been developed to explain how decision-making processes can be shaped that are based on theoretical considerations [21, 22, 25]. For instance, the ethicist Bernard Rollin distinguishes between two models that veterinarians can refer to during the care of their patient: first, veterinarians can act as the “garage mechanic” or second, take the role of “paediatrician” and prioritize the patient’s interest [25]. Our patient-focused DEO has important similarities to Rollin’s [25] paediatrician model. While Rollin argues, as does Coghlan [22], that veterinarians should uphold the role as “paediatricians” and advocate accordingly, we do not promote a particular view here, and instead focus on understanding how veterinarians *actually* shape their decision-making, rather than proposing how they *should* act.

Our results provide novel insights into professionals’ strategies in relation to the development-oriented and client-empathetic DEOs. As expected, the high level of agreement in statements related to the patient-oriented orientation indicates that this orientation is highly important to the veterinarians. Agreement in the client-empathetic statements were even higher than in the patient-focused statements, indicating the importance of being emotionally supportive towards the client, as well as considering the patients’ interests. The identification of a development-oriented DEO perhaps reflects the rapid advancement of small animal practice encouraging (or forcing) veterinarians to upskill and offer the latest treatments to their clients [1]. Our results revealed that veterinarians’ attitudes towards rapid developments in diagnostics and treatment are quite varied in all three countries.

The correlations between the DEOs are relatively modest with positive associations between the client-empathetic, development-oriented, and patient-focused orientations (in all cases below r = 0.300). In addition to the main aim of identifying the four DEOs, we were interested in whether these orientations varied between veterinarians in the different countries. Overall, the client-empathetic, development-oriented and client-devolved DEOs varied at a statistically significant level between the three countries studied here. However, these differences between countries are actually rather modest and should be explored for a wider range of countries.

In addition, we aimed to identify whether factors specific to practice structure and socio-demographics within countries could explain any differences in DEOs, and to what extent. As corporate business models reportedly encourage veterinarians to focus on business aspects and generating revenue [32], we expected veterinarians in such environments to be less client-devolved or client-empathetic. Interestingly this proved not to be the case as results of multivariate regression analyses indicate that business type has no effect on whether veterinarians adopt either client-empathetic or client-devolved DEOs in the UK and Denmark. However, this was not the case for UK veterinarians, where those working in corporate practices or clinics were significantly less patient-focused than their colleagues working in independently owned practices or clinics. As Nicol [32] discusses, if veterinarians are asked to adhere to standardized treatment protocols, for example, this will impinge on their freedom to make independent clinical decisions bespoke to their patients. The potential for such policies or financial targets to influence veterinarian’s autonomy and DEOs within the context of corporate practice merits further investigation. Conversely, working as part of a corporate practice or clinic can bring benefits in terms of flexibility towards client’s expectations, a high budget for equipment purchase, regular work schedules for employees and good exit strategies for retirees [30, 32].

Further, we identified that significantly more Austrian veterinarians work in self-employment (79.4%) and privately-owned practices (95.1%) than their Danish and UK colleagues. It is reasonable to assume that those veterinarians are economically strongly dependent on their clients and would put more emphasis on meeting their clients’ emotional needs and demands. We therefore expected that Austrian veterinarians would be more client-empathetic in their DEO compared to UK and Danish veterinarians. Unexpectedly this proved not be the case, as Austrian veterinarians were significantly less client-empathetic than their colleagues in the UK and Denmark.

Interestingly, our analyses revealed that, with the exception of UK veterinarians working in different business types, the patient-focused DEO was not affected by socio-demographic or practice-specific factors in any country, nor did it differ between veterinarians in the three countries. This is likely because consideration of patient’s interests is a core principle shared by veterinarians across all countries. Interestingly, ordinal regression analysis indicated that having a patient-focused DEO did not influence the frequency with which a veterinarian would discuss pet health insurance with owners who do not have it. This could simply be a consequence of the minimal variation in veterinarians’ patient-focused DEO. Further investigation is merited to determine whether differences in considerations of the patient’s interest might arise in practice-specific situations where the conflicting values that we have described in this paper are activated.

There is no doubt that many advancements in diagnostic and treatment modalities have been made in modern small animal practice, similar to those offered in human medicine [1]. Veterinarians who participated in an Austrian focus group study stated that such developments not only simplify working processes and motivate them in their daily work life, but also stimulate them to continuously educate themselves and make use of the new techniques [1]. Based on this, we hypothesized the existence of the development-oriented DEO. Results of the present study suggest that UK veterinarians are less development-oriented compared to their Danish and Austrian colleagues. Although the country difference was very modest, we were surprised by this in view of the high level of development and specialization of the veterinary profession in the UK, which has six university veterinary hospitals, a large number of veterinary specialists [45], and a high prevalence of corporate business models [28] and pet health insurance. It is possible that veterinarians in the UK are simply more used to the requirement for continuous professional development, and hence more likely to take the advanced diagnostic and treatment modalities for granted. This may explain why UK veterinarians were less likely to report that advanced methods should be implemented compared to Danish and Austrian veterinarians.

Many empirical studies have found that gender plays a role in ethically challenging situations and veterinarians’ level of moral stress [8, 9, 46–49], and so we expected gender to influence DEOs. Surprisingly, the only significant finding in our study in relation to gender was that male veterinarians had a greater tendency towards the development-oriented DEO than their female colleagues. While gender did not appear to play a decisive role in how veterinarians shape their decision-making and deal with conflicting interests, we recommend further studies to explore this. For instance, it would be relevant to look at whether the interactions between gender and the DEOs affect specific clinical decisions.

In contrast to gender, work experience and age have an impact on DEOs. In general, more work experience and increased age result in a greater tendency towards the client-empathetic DEO. Here, our findings contrast with results of a survey conducted with North American veterinarians on ethical conflicts and moral distress [46]: on the question of whether veterinarians lose compassion for clients over the course of their practice, the majority of North American veterinarians tended to answer this question with “yes” (31.4%) or “sometimes” (43.2%). Such findings support the idea that compassion fatigue occurs in the course of veterinarians’ work life [50]. Relatedly the study by Orcutt and colleagues [50] on veterinary technicians, veterinarians and students in the US, found that veterinarians with many years of experiences in practice do not perform with the same enthusiasm as when they first started in the field. However, we identified an opposite trend, suggesting that experienced veterinarians are more aware of the importance of empathic relationships with clients. Empathy not only increases client satisfaction and positive recognition of veterinarians’ work, but can also facilitate the integration of the patient’s interests into the decision-making processes [1]. Arguably, a veterinarian with many years of experience will likely have long-standing relationships with many of their clients, predisposing them towards client-empathetic decision ethics.

In addition, we found that veterinarians’ work experience and age also influence the prevalence of the development-oriented DEO. Less experienced and younger veterinarians are more likely to promote the advancement of small animal practice compared to their more experienced colleagues. This may be because they have been more recently trained at well-equipped university veterinary hospitals and are therefore more used to advanced technology and innovative methods.

Another aim of our study was to explore the extent to which the different orientations influence the clinical consultation processes. A number of publications indicate that client’s financial limitations are a common and challenging aspect of veterinarians’ daily work life [8–10, 46]. The existence of pet health insurance reduces the likelihood of clients being unable to afford to pay for necessary diagnostic tests and treatment. Therefore, we found it useful to explore the connection between DEOs and how frequently veterinarians discuss the possibility of health insurance for dogs and cats with clients who do not have it.

We expected that the development-oriented DEO in particular might correlate positively with the frequency with which insurance was discussed, if veterinarians aim to offer the highest standard of care, potentially involving the use of expensive technologies or treatment modalities. This was the case with the Danish and UK veterinarians. Interestingly, regression analyses indicated that UK veterinarians with higher client-empathetic scores were also more likely to discuss insurance, which is not surprising if veterinarians are empathetic to the stressful effects of financial limitations on clients’ wellbeing. An interview study with twelve dog owners [3] found that clients with high levels of emotional attachment to their animal would make monetary sacrifices to be able to give the animals the best possible care [3]. It seems likely that veterinarians who strongly empathize with a client in a difficult situation will also be adversely affected themselves by having to ask for payment–thus treating insured pets likely also reduces the veterinarians’ level of stress.

We identified that Austrian veterinarians less frequently discuss health insurance than their Danish and UK colleagues. These differences are probably influenced by the varying prevalence of insured animals across European countries. In the UK, in 2019, around 57% of dogs and 37% of cats were insured [51, 52]. In 2016, a Danish study among 600 dog owners identified that 39% had health insurance for their dog [53]. In contrast, the pet insurance market in Austria is relatively small and the estimated number of insured animals is far below 10% [26]. Further, in an Austrian focus group study with veterinarians [1] the topic of health insurance was not very prominent during the discussions, suggesting that health insurance has little impact on their everyday working life and consequently on clinical decision-making processes. This is also evident in regard to results of the regression analysis and the identification of possible correlation of the frequency of discussion about health insurance and the four DEOs. Even though the results do not provide information in regard to the causality, in both analyses, no significant correlation or effects were found for Austria.

Although this transnational study includes three countries for a comprehensive investigation of veterinarians’ DEOs, the study is subject to several limitations. Collaborating with small animal associations in each country enabled us to invite veterinarians who were only or mainly working with small animals. However, not all small animal veterinarians are members of a veterinary association, leading to selection bias especially in the UK and Austria, where coverage error was around 30%. Further, the response rates were quite low in all three countries, perhaps partly because the study was undertaken during the COVID-19 pandemic. A possible consequence of low response rates is non-response bias, where respondents that participate in a study deviate systematically from respondents who were invited but declined to participate. It is not possible to determine whether the results exhibit non-response bias, but we note that there was very good socio-demographic coverage in the three samples. Through regression analysis, we tried to account for possible non-response bias by socio-demographic adjustment (through regression analysis) of the main findings, but this does not fully address the problem. In comparison to Denmark and UK, the small response rate resulted in a quite small sample size in Austria, which means that the ability to detect significant differences within Austria was hampered.

By means of PCA and subsequent confirmatory factor analysis we were able to identify the four DEOs in Austria, Denmark and the UK. Further, the ordinal alpha coefficients suggest that the four DEOs were identified with acceptable levels of precision for early stages of research [44]. However, since all ordinal alpha coefficients were below 0.80, and two were below 0.70 future research should consider improving the internal consistency by including extra items for each of the four orientations. Since the DEOs were developed on the basis of empirical findings from several countries, we assume that they will be widely applicable, but recommend further studies to examine potential differences as a result of cultural traditions, legal frameworks, and forms of business. As this study was the first of its kind, we made use of on an exploratory approach where multiple tests of association were conducted. This, however, increases the risk of false discoveries (type 1 errors). For this reason, all bivariate tests of associations were adjusted. Nevertheless, we recommend future research in this field to be more hypothesis-driven to avoid type 1 errors.

This study forms part of a larger body of work on ethical challenges in modern small animal practice. By following a mixed-methods approach, the conceptualization of the four hypothesized DEOs is mainly based on qualitative interview data from an Austrian focus group study [1] and a literature review of various empirical studies [3, 4, 8, 9, 11, 12, 31, 33–37] within the field of small animal practice. It is important to note that our proposed set of veterinarian’s decision strategies is not exhaustive, nor is our suggestion the only way to approach the question of how veterinarians shape their clinical decision-making. For example, we are aware that it would be possible to focus more on the veterinarian’s income, or the clients’ cognitive needs in relation to their level of information and knowledge (in addition to emotional needs) in order to frame and conceptualize veterinary decision-making strategies. Hence, future research should focus on whether other aspects could be considered and included that possibly modify DEOs in our suggested set of strategies. Further, we highly recommend future research to investigate the extent to which the DEOs impact specific diagnostic or treatment recommendations in various situations. Of particular interest is the question of how DEOs influence the way in which veterinarians manage situations in which an uninsured client cannot afford to pay for treatment, in contrast to a fully insured client facing similar treatment choices. Additionally, we see a need to further explore positive as well as negative aspects related to health insurance for dogs and cats, and how veterinarian’s attitudes towards health insurance might be affected by the DEOs.

## Conclusions

Based on results of our transnational study, we identified four DEOs, which describe ways in which veterinarians approach conflicting expectations and concerns in modern small animal practice: First, a client-empathetic DEO that prioritizes empathy with clients’ emotional well-being and personal circumstances during decision-making processes. Second, a development-oriented DEO that prioritizes veterinarians’ own desires to advance small animal medicine. Third, a patient-focused DEO where the patient’s interests are at the center of the decision-making processes. And fourth, a client-devolved DEO that reflects a preference to leave the final decisions to the client, without having to consider the client’s personal problems (e.g. financial issues). Results indicate that the patient-focused and client-empathetic DEO were widely shared by Austrian, Danish and UK veterinarians. The DEOs were associated with practice-specific factors, and also influenced the frequency with which health insurance was discussed with clients. With increasing experience and greater age for example, veterinarians tended to be more client-empathetic. In the same way, Danish and UK veterinarians who strongly empathize with the client, or are motivated to advance veterinary medicine, more frequently discuss the opportunity of health insurance. Interestingly however, we found no evidence that gender plays a decisive role in determining a preference for a particular DEO.

## Supporting information

S1 Appendix English version of the questionnaire. (PDF)

S1 Table Socio-demographic and practice-specific aspects for the whole study population and each sub-population from Austria, Denmark and UK. (PDF)

S2 Table Decision ethics orientations scores across socio-demographic and practice-specific factors by countries. (PDF)
