# Supplementary material for: Managing conflicting ethical concerns in modern small animal practice—A comparative study of veterinarian’s decision ethics in Austria, Denmark and the UK
Source: PLoS One. 2021 Jun 18;16(6):e0253420. doi: 10.1371/journal.pone.0253420 (PMC8213188; doi:10.1371/journal.pone.0253420)
Supplement: S1 Appendix — (PDF) [file pone.0253420.s001.pdf]

Transnational Questionnaire Study on “Modern small animal practice”

Introduction and background:

Thank you for participating in this survey. The **purpose** of the survey is to gather opinions from **veterinarians in Austria, Denmark and the UK on developments in small animal practice,** and to explore how these opinions might **influence decision-making**. **The questionnaire consists of three sections** and it should take **approximately 20 minutes to complete**. Your answers will help us to understand the ethical dilemmas faced by veterinary professionals, and how they are handled. The results will be widely disseminated through the veterinary press in all three countries, and publication in international journals.

The study is part of a project funded by the FWF (Austrian Science Fund) and the University of Copenhagen. It is being conducted through the University of Veterinary Medicine, Vienna, University of Copenhagen and University of Glasgow. The questionnaire was approved by ethics commitees and collaborating organisations in Austria (VÖK), Denmark (DVA) and the UK (BSAVA).

Completion of the questionnaire is **voluntary** and you are **able to exit the survey at any point**. **Your responses will be anonymous, and not linked to any information that can be traced back to you**. However, if you would like a short summary of the results of the study, you can provide your e-mail address via a separate survey at the end of the questionnaire.

Thank you for your contribution!

SECTION A:

Socio-demographic questions

Initially, we kindly ask you to provide some information on demographics, your career, and professional working environment.

1: Please enter your current age in years from the drop-down menu. *(drop-down menu from <23 - 100 years + "prefer not to say")*

_________ years old.

2: Please indicate your gender.

Male	
Female	
Non-binary	
Prefer not to say	

3: Do you work in other veterinary field(s) as well as small animal practice (e.g. production animal)?

Yes	
No	

3.1: Please indicate which one(s): *(logic question; only if "yes" was chosen in question 3)*

Tick all that are relevant.

Production animal	
Equine	
Laboratory animal	
Government	
Meat inspection and food safety	

Zoo animal	
Other	

4: How long have you been working as veterinarian? *(drop-down list from <0,5 - 80 years)*

___________ years.

5: What is your current employment status? *(multiple choice)*

Self-employed full-time	
Self-employed part-time	
Employed full-time	
Employed part-time	
Retired, but still professionally active	
Retired	
Other	

5.1: How many years have you been retired? *(drop-down list from <0,5 – 50 years) (logic question; only if "retired" under question 5)*

___________ years.

6: In which of the following do you currently work as small animal veterinarian? *(multiple choice)*
6: In which of the following did you last work as small animal veterinarian? *(logic question; formulation for answer option "retired" under question 5)*

For UK:

Independently owned general practice	
Corporate owned general practice	

Independently owned referral practice	
Corporate owned referral practice	
University hospital	
Shelter and/or charity (as an employee)	
Other	

For Austria:

Private Tierarztpraxis	
Vergesellschaftete Tierarztpraxis (z.B. Anicura)	
Private Tierklinik	
Vergesellschaftete Tierklinik (z.B. Anicura)	
Universitätsklinik	
Tierheim und/oder Wohltätigkeitsorganisation (im Angestelltenverhältnis)	
Andere	

For Denmark:

Independently owned small animal practice	
Corporate owned small animal practice	
Independently owned small animal referral hospital	
Corporate owned small animal referral hospital	
University hospital	
Shelter and/or charity as an employee	
Other	

7: In which region(s) do you currently work as small animal veterinarian? *(multiple choice)*

7: In which of the following regions did you last work as small animal veterinarian? *(logic question; formulation for answer option "retired" under question 5)*

For UK:

Scotland	
Northern Ireland	
Wales	
England - North East	
England - North West	
England - Yorkshire and Humberside	
England - West Midlands	
England - East Midlands	
England - South West	
England - South East	
England - Greater London	
Prefer not to say	

For Austria:

Western Austria (Vorarlberg, Tyrol, Salzburg, Upper Austria)	
Eastern Austria (Lower Austria, Vienna, Burgenland)	
South Austria (Carinthia, Styria)	
Prefer not to say	

For Denmark:

Region Hovedstaden	

Region Midtjylland	
Region Nordjylland	
Region Sjælland	
Region Syddanmark	
Prefer not to say	

8: How many small animal veterinarians work in the practice/clinic/hospital (including yourself)? *(drop down list from 1 - >15 veterinarians)*
8: How many small animal veterinarians worked in the last practice/clinic/hospital where you worked before you retired (including yourself)? *(logic question; formulation for answer option "retired" under question 5)*

___________ veterinarian(s).

In case of a second working place: ___________ veterinarian(s).
In case of a third working place: ___________ veterinarian(s).

___________ Tiermediziner(innen).
Im Falle eines zweiten Arbeitsplatzes: ___________ Tiermedizinier(innen).
Im Falle eines dritten Arbeitsplatzes: __________ Tiermediziner(innen).

9: Are you involved in daily management in the practice/clinic/hospital?
9: Were you involved in daily management in the practice/clinic/hospital?
(logic question; formulation for answer option "retired" under question 5)

Yes	
No	

9.1: What percentage of your working time do you spend on management? *(logic question; only if respondents choose "yes" under question 9)*
9.1: What percentage of your working time did you spend on management? *(logic question; formulation for answer option "retired" under question 5)*

1. 1-9%
2. 10-19%
3. 20-29%
4. 30-39%
5. 40-49%
6. 50-59%
7. 60-69%
8. 70-79%
9. 80-89%
10. 90-99%
11. 100%

10: Is your income dependent on the income of the practice/clinic/hospital? *(logic question: only if "employed full-time", "employed part-time" and "other" was chosen under question 5)*
10: Was your income dependent on the income of the practice/clinic/hospital? *(logic question: only if "retired" was chosen under question 5)*

Yes	
No	

11: Are you personally allowed to discount client fees without permission? *(logic question: only if "employed full-time", "employed part-time" and "other" was chosen under question 5)*
11: Were you personally allowed to discount client fees without permission? *(logic question: only if "retired" was chosen under question 5)*

Yes	
No	

12: Do you have any of the following qualifications in addition to your veterinary degree?

Tick all that are relevant.

For Austria:

Fachtierarzt	
Diplomate (z.B. European oder American College)	
Doctor of veterinary medicine	
PhD	
Masters	
Other	
I did not undertake any of the above qualifications.	

For UK:

Diploma (e.g RCVS, European or American College)	
Masters	
PhD	
Other	
I did not undertake any of the above qualifications.	

For Denmark:

Fagdyrlæge/Øjenpanel dyrlæge/eller anden tilsvarende efter-videreuddannelse	
Master i familiedyrsvidenskab	
PhD	
Dansk specialdyrlæge godkendt af Fødevarestyrelsen	
EBVS eller ABVS Diplomat	

Anden<br>Other	
Ingen af ovenstående<br>None of the above	

12.1: In which area are you specialized? Tick all that are relevant.

(logic question: only if respondents chose under question 11: AUSTRIA: Fachtierarzt; Diplom (z.B. European oder American College); Master; UK: Master; Diploma (RCVS, ECVS or ACVS board certification); DENMARK: Fagdyrlæge/VetCEE/Eyepanelist/RCVS certificate; Master, Companion Animal Clinical Sciences, Danish National specialist authorisation by Fødevarestyrelsen, Diploma (ECVS or ACVS board certification))

Anesthesia	
Behaviour	
Birds / exotics / laboratory animal / zoo medicine	
Cardiology	
Diagnostic imaging	
Dentistry	
Dermatology	
Internal medicine	
Neurology	
Oncology	
Ophthalmology	
Reproduction	
Sports medicine and rehabilitation	
Surgery (soft-tissue)	
Surgery (orthopaedics)	
Other	

13: What percentage of your cases are dogs?

13: What percentage of your cases were dogs? *(logic question: only if "retired" was chosen under question 5)*

12. 100 %
13. 90–99 %
14. 80–89 %
15. 70–79 %
16. 60-69 %
17. 50-59 %
18. 40-49 %
19. 30-39 %
20. 20-29 %
21. 10-19%
22. 1-9 %
23. I do not treat dogs. *or* I did not treat dogs.

14: What percentage of your cases are cats?

14: What percentage of your cases were cats? *(logic question: only if "retired" was chosen under question 5)*

1. 100 %
2. 90–99 %
3. 80–89 %
4. 70–79 %
5. 60-69 %
6. 50-59 %
7. 40-49 %
8. 30-39 %
9. 20-29 %

10. 10-19%
11. 1-9 %
12. I do not treat cats. *or* I did not treat cats.

15: Which of the following diagnostic / therapeutic options are or were available in your clinic/practice/hospital where you work(ed)?

15: Welche der folgenden diagnostischen und therapeutischen Möglichkeiten stehen bzw. standen in Ihrer Klinik/Praxis zur Verfügung in der Sie arbeiten bzw. arbeiteten?

Screen-film radiography	
Digital radiography	
Ultrasound	
Echocardiography	
Electrocardiography	
Endoscopy	
Laparoscopy	
Thoracoscopy	
Arthroscopy	
In-house laboratory	
Dental equipment/unit	
MRI scanner	
CT scanner	
Linear accelerator	
None of the above	

SECTION B:

B.1: Advances in small animal practice

1.1: The second part of the questionnaire focuses on some aspects of advanced small animal practice. We would like to know the extent to which you agree with the following statements.

1= strongly disagree; 2 = disagree; 3 = somewhat disagree; 4 = neutral (neither agree nor disagree); 5 = somewhat agree; 6 = agree and 7 = strongly agree

		1	2	3	4	5	6	7
1	Veterinary medicine should offer the same **diagnostic** options as human medicine.							
2	Veterinary medicine should offer the same **treatment** options as human medicine.							
3	It is important for the veterinary profession to keep developing innovative methods, even though it is impossible to predict possible complications.							
4	Sometimes veterinarians need to risk potential harm to the individual patient to advance small animal medicine.							
5	It is important to promote the advancement of small animal medicine for future patients.							
6	Veterinarians working in a university setting should have a greater responsibility to advance veterinary medicine than their colleagues working in other clinics or practices.							

B.2: Factors related to the patient, the client and veterinarian's professional environment

2.1: With reference to your daily work life, to what extent do you agree with the following statements?

2.1: In the context of your previous working life, to what extent do you agree with the following statements? *(logic question: only if "retired" was chosen under question 5)*

1= strongly disagree; 2 = disagree; 3 = somewhat disagree; 4 = neutral (neither agree nor disagree); 5 = somewhat agree; 6 = agree and 7 = strongly agree *(items will be presented on two pages → 9 items per page)*

		1	2	3	4	5	6	7
1	The patient is my first priority when I make medical decisions.							
2	I have no problem with making compromises in patient care if the clients have financial limitations.							
3	I am unhappy if I have to make any compromise in relation to the patient's care.							
4	I am willing to provide palliative care for up to 3 days even if euthanasia is indicated, if the clients are not ready to part with their animal immediately.							
5	I advise the client about all possible treatment options, but it is up to the client to decide between them.							
6	It is important to empathise with the client's emotions in the decision-making process.							
7	I try to convince clients to give patients the best possible care, even if I know this will be a financial burden for them.							
8	I empathise with clients who cannot part with their animal.							
9	It is important to include client's interests in the decision-making process, even if this means providing suboptimal treatment to the patient.							
10	It is important to make decisions that are in the patient's best interests, even if I might lose the owner as client.							
11	It is my responsibility to find the best possible solution for the patient <u>and</u> the client.							

12	It is important to act not only professionally, but also to be emotionally supportive to the client.							
13	I do not make the decisions - the client has to make them for the patient.							
14	I empathise with lonely and elderly clients in my decision-making processes.							
15	In situations where my opinion differs from that of the client, I will ultimately let the client decide.							
16	It is more important for me to act in the best interest of the patient than of the client.							
17	It is important to empathise with clients' family and social situation in the decision-making.							
18	I only make use of new techniques if I know they will directly benefit the individual patient.							

B.2.1: Owner-compliance: Acceptance of veterinary medical advice

2.2: On average, how often do clients want to pursue treatment beyond your recommendation? *(single choice answer option)*
2.2: On average, how often did clients want to pursue treatment beyond what you had recommended? *(logic question: only if "retired" was chosen under question 5)*

Never	
Less than once a month	
1-2 times a month	
3-4 times a month	
5-10 times a month	
More than 10 times a month	
I don't know	

2.3: To what extent do you think the following factors influence clients' desire to pursue further therapy beyond your recommendation? *(logic question: only if "less than once per month"; "1-2 times a month"; "3-4 times a month"; "5-10 times a month" and "I don't know" was chosen under question 2.2)*

2.3: To what extent do you think the following factors influenced clients' desire to pursue treatment beyond your recommendation? *(logic question: only if "less than once per month"; "1-2 times a month"; "3-4 times a month"; "5-10 times a month" and "I don't know" was chosen under question 2.2 and only if "retired" was chosen under question 5)*

1= not at all; 2 = slightly; 3 = moderately; 4 = relatively strong; 5 = very strong and 6 = I don't know

		1	2	3	4	5	6
1	Having a strong emotional bond with their animal.						
2	Medical information they obtained using internet resources.						
3	Belief that it is in the best interest of their animal.						
4	Having had, or heard of, a postive experience with another animal going through the treatment.						
5	Having had a positive experience with family or friends going through the same kind of treatment.						
6	Positive reports about the treatment in the press and social media.						
7	Having obtained a second opinion.						
8	The patient being insured.						

2.4: On average, how often do clients refuse further treatment that you recommend?

2.4: On average, how often did clients refuse further treatment that you had recommended? *(logic question: only if "retired" was chosen under question 5)*

Never	
Less than once per month	
1-2 times per month	
3-4 times per month	
5-10 times per month	
More than 10 times per month	
I don't know	

2.5: To what extent do you think the following factors influence clients when they refuse further treatment that you recommend? *(logic question: only if "less than once per month"; "1-2 times a month"; "3-4 times a month"; "5-10 times a month" and "I don't know" was choen under question 2.2)*

2.5: To what extent do you think the following factors influenced clients when they refused further treatment that you recommended? *(logic question: only if "less than once per month"; "1-2 times a month"; "3-4 times a month"; "5-10 times a month" and "I don't know" was choen under question 2.2 and logic question: only if "retired" was chosen under question 5)*

1 = not at all; 2 = slightly; 3 = moderately; 4 = relatively strong; 5 = very strong and 6 = I don't know

		1	2	3	4	5	6
1	Limited financial resources.						
2	A weak emotional bond with the animal.						
3	Limited time resources to care for the animal.						
4	Belief that the treatment would not be in the animal's best interests.						

5	Having had, or heard of, a bad experience with another animal going through the treatment.						
6	Having had a bad experience with family or friends going through the same kind of treatment.						
7	Negative reports about the treatment in the press and social media.						
8	Clients' desire to consult another (specialist) veterinarian.						
9	Fear that the animal will not tolerate treatment e.g. aneasthesia, chemotherapy.						

B.3: Health insurance for dogs and cats in veterinary practice

3.1: To what extent do you agree with the following statements?

1= strongly disagree; 2 = disagree; 3 = somewhat disagree; 4 = neutral (neither agree nor disagree); 5 = somewhat agree; 6 = agree; 7 = strongly agree and 8 = I don't know

	**Coverage by health insurance…**		1	2	3	4	5	6	7	8
1	▪ reduces stress for veterinarians as financial limitations are less important in treatment decisions.									
2	▪ should be mandatory for dog and cat owners to ensure that treatment is never determined by financial limitations.									
3	▪ influences the use of diagnostic tests and type of treatment due to specific restrictions in the insurance policy.									
4	▪ can lead to over-use of diagnostic tests as financial limitations are less likely.									
5	▪ can lead to overtreatment as financial limitations are less likely.									
6	▪ is not necessary.									

3.2: What percentage of the dogs you treat are insured?

3.2: What percentage of the dogs you treated were insured? *(logic question: only if "retired" was chosen under question 5)*

1. I do not treat dogs. *or* I did not treat dogs.
2. None.
3. <10%
4. 11-20%
5. 21-30%
6. 31-40%
7. 41-50%
8. 51-60%
9. 61-70%
10. 71-80%
11. 81-90%
12. 91-100%
13. I don't know.

3.3: What percentage of the cats you treat are insured?

3.3: What percentage of the cats you treated were insured? *(logic question: only if "retired" was chosen under question 5)*

1. I do not treat cats. *or* I did not treat cats.
2. None.
3. <10%
4. 11-20%
5. 21-30%
6. 31-40%
7. 41-50%
8. 51-60%

9. 61-70%
10. 71-80%
11. 81-90%
12. 91-100%
13. I don’t know.

3.4: How often do you discuss the possibility of health insurance with owners who do not have it?
3.4: How often did you disucss the possibility of health insurance with owners who did not have it? *(logic question: only if “retired” was chosen under question 5)*

Never	
Occasionally	
Frequently	
Always	
I don’t know	

B.4.: The use of Facebook for veterinary clinics/practices/hospitals and evaluations on webpages and/or social media platforms

4.1: Does the clinic/practice/hospital where you work have an active Facebook page? *(single choice)*
4.1: Did the clinic/practice/hospital where you worked have an active Facebook page? *(single choice) (logic question: only if “retired” was chosen under question 5)*

Yes	
No	
I don’t know	

4.2: To what extent do you agree with the following statements?

1= strongly disagree; 2 = disagree; 3 = somewhat disagree; 4 = neutral (neither agree nor disagree); 5 = somewhat agree; 6 = agree; 7 = strongly agree and 8 = I don’t know

	**Having a Facebook page for the practice/clinic/hospital…**	1	2	3	4	5	6	7	8
1	▪ gives clients an insight into what happens with the patient in the practice/clinic/hospital.								
2	▪ is of no relevance.								
3	▪ is a vital way to recruit new clients.								
4	▪ is expected by clients.								
5	▪ is an important way of communicating for veterinarians and their clients.								

4.3: On average, how often do you find negative comments from clients about your veterinary service on webpages and/or social media platforms? *(single choice)*

4.3: On average, how often did you find negative comments from clients about your veterinary service on webpages and/or social media platforms? *(single choice) (logic question: only if “retired” was chosen under question 5)*

Never	
Less than once per month	
1-2 times per month	
3-4 times per month	
5-10 times per month	
More than 10 times per month	
I don’t know	
Prefer not to say	

4.4: What are the negative comments about? Tick all that are relevant. *(logic question: only if "less than once per month"; "1-2 times a month"; "3-4 times a month"; "5-10 times a month" and "I don't know" was choen under question 4.3; multiple choice)*

**Clients…**	
▪ having to wait too long.	
▪ disagreeing with my medical advice.	
▪ complaining about the handling of the patient.	
▪ complaining about the cost of treatment	
▪ complaining about specific staff member(s).	
▪ complaining about the lack of technical euqipment in the practice/clinic/hospital.	
▪ complaining about communication.	
I don't know.	
Other	

4.4: What were the negative comments about? Tick all that are relevant. *(logic question: only if "less than once per month"; "1-2 times a month"; "3-4 times a month"; "5-10 times a month" and "I don't know" was choen under question 4.3; multiple choice and only if "retired" was chosen under question 5 (Section A))*

**Clients…**	
▪ had to wait too long.	
▪ disagreed with my medical advice.	
▪ complained about the handling of the patient.	
▪ complained about the cost of treatment	
▪ complained about specific staff member(s).	
▪ complained about the lack of technical euqipment in the practice/clinic/hospital.	
▪ complained about communication.	
I don't know.	
Other	

4.5: How do you deal with the negative feedback? Tick all that are relevant.

(logic question: only if "less than once per month"; "1-2 times a month"; "3-4 times a month"; "5-10 times a month" and "I don't know" was choen under question 4.3)

I am not in charge of dealing with negative feedback.	
I ignore the feedback.	
If possible I delete the feedback or try to get it deleted.	
If possible I reply to any feedback that I feel is unfair.	
If possible I reply to every piece of negative feedback.	
If the clients come back to me, I discuss the negative feedback with them.	
Other	

4.5: How did you deal with negative feedback? Tick all that are relevant.

(logic question: only if "less than once per month"; "1-2 times a month"; "3-4 times a month"; "5-10 times a month" and "I don't know" was choen under question 4.3; multiple choice and only if "retired" was chosen under question 5 Section A)

I was not in charge of dealing with negative feedback.	
I ignored the feedback.	
If possible I deleted the feedback or tried to get it deleted.	
If possible I replied to any feedback that I felt was unfair.	
If possible I replied to every piece of negative feedback.	
In cases where the clients came back to me, I talked to them about their negative feedback.	
Other	

B.4.1: Client's use of internet resources

4.6: How many of your clients do you think use internet resources to find medical information prior to consultations with you?

4.6: How many of your clients do you think used internet resources to find medical information prior to consultations with you? *(logic question: only if "retired" was chosen under question 5)*

1. None
2. 1–19 %
3. 20–39 %
4. 40–59 %
5. 60–79 %
6. 80–100 %
7. I don't know

4.7: To what extent do you agree with the following statements?

1= strongly disagree; 2 = disagree; 3 = somewhat disagree; 4 = neutral (neither agree nor disagree); 5 = somewhat agree; 6 = agree; 7 = strongly agree and 8 = I don't know

	**Clients' use of internet resources…**	1	2	3	4	5	6	7	8
1	▪ results in greater acceptance of advanced diagnostics and treatments.								
2	▪ results in greater expectations of advanced diagnostics and treatments.								
3	▪ improves the discussion about diagnostic and treatment options as the clients have greater knowledge.								
4	▪ can lead to situations where clients are better informed than I am.								
5	▪ causes clients to form strong opinions so that veterinarians have to justify their diagnostic and therapeutic steps.								
6	▪ creates a wrong impression of modern small animal practice.								

4.8: How often do clients question your professional advice based on information they obtained from internet resources?

4.8: How often did clients question your professional advice based on information they obtained from internet resources? *(logic question: only if "retired" was chosen under question 5)*

Never	
Occasionally	
Frequently	
Always	
I don't know	

SECTION C:

Case Vignettes

Finally, we would like to present you with two case vignettes related to the patient, the client and veterinarians' working environment. In each case, please place ("drag and drop") each of the proposed reasons into the category that best reflects how important that reason was in making your decision.

C.1. Differential treatment of dogs and cats with cancer

(Different formulations of case vignettes: The study population will be divided into two groups. The first group includes all respondents working as ***oncologists (Group 1)****. The second group includes all veterinarians working as* ***general practitioners or in other fields of specialization (Group 2)****.*

Experimental approach: The study population (Group 1 and 2) will be divided into two groups each.

Group 1a. and 2a. will be given the case vignette, where the patient is a CAT.

Group 1b. and 2b. will be given the case vignette, where the patient is a DOG:

Group 1.a: *Half of oncologists, who will be given* ***case example 1.1*** *(patient is a cat)*

Group 1.b: *Half of oncologists, who will be given* ***case example 1.2.*** *(patient is a dog)*

Group 2.a: *Half of respondents in Group 2 (generalists and other specialists) will be given* ***case example 1.3*** *(patient is a cat)*

Group 2.b: *The other half of Group 2 will be given* ***case example 1.4*** *(patient is a dog)*

1.1: Cancer treatment: Cat with *a feline injection-site sarcoma* (GROUP 1.a | oncologists)

An owner consulted her general practitioner about her twelve-year old cat that had developed a mass on the back of the neck four months after being vaccinated. Your colleague suspected an injection site sarcoma. Staging showed no evidence of metastatic disease and radical surgical excision was performed. Histopathology confirmed the diagnosis, but identified dirty margins, and the patient has now been referred to you, to discuss the option of radiotherapy with the client. Although radiotherapy would reduce the risk of recurrence, the client is unsure, and asks you: "What would you do, if it was your cat?"

How would you advise the client? *(single choice)*

1	I would recommend radiotherapy.	
2	I would recommend against radiotherapy.	
3	I would not make any recommendation regarding radiotherapy.	
4	Other	

SUB-QUESTIONS:

IF YOU CHOSE OPTION 1:

Please 'drag and drop' each of the following reasons into the category that best reflects how important it was in making your decision. Multiple usage of the individual fields possible.

Reason 1	There is scientific evidence that radiotherapy will benefit the patient.
Reason 2	I have had positive experiences with radiotherapy in cats.
Reason 3	The client wants to try anything that might benefit the cat.
Reason 4	I think that every possible treatment option should be tried.

**Very important reason**	**Important reason**	**Less important reason**	**Not important at all**	**I don't know**

IF YOU CHOSE OPTION 2:

Please 'drag and drop' each of the following reasons into the category that best reflects how important it was in making your decision. Multiple usage of the individual fields possible.

Reason 1	I am not aware of scientific evidence that radiotherapy will benefit the patient.
Reason 2	I do not believe that radiotherapy will extend the cat's life.
Reason 3	The cat's age.
Reason 4	I think that cats do not tolerate the side-effects.
Reason 5	The practice/clinic/hospital where I work does not offer radiotherapy.

**Very important reason**	**Important reason**	**Less important reason**	**Not important at all**	**I don't know**

IF YOU CHOSE OPTION 3:

Please 'drag and drop' each of the following reasons into the category that best reflects how important it was in making your decision. Multiple usage of the individual fields is possible.

Reason 1	I never answer the question "What would you do, if it was your animal?"
Reason 2	It is my responsibility to offer all possible treatment options and adverse effects, but not to decide for the client.

**Very important reason**	**Important reason**	**Less important reason**	**Not important at all**	**I don't know**

1.2: Cancer treatment: Dog with *a soft tissue sarcoma* (GROUP 1.b | oncologists)

An owner consulted her general practitioner about her twelve-year old mixed-breed dog that has developed a firm mass on the back of the neck. Your colleague suspected a soft tissue sarcoma (STS). Staging showed no evidence of metastatic disease and radical surgical excision was performed. Histopathology confirmed a STS grade III with dirty margins, and the patient has now been referred to you, to discuss the option of radiotherapy with the client. Although radiotherapy would reduce the risk of recurrence, the client is unsure and asks you: “What would you do, if it was your dog?”

How would you advise the client? *(single choice)*

1	I would recommend radiotherapy.
2	I would recommend against radiotherapy.
3	I would not make any recommendation regarding radiotherapy.
4	Other

SUB-QUESTIONS:

IF YOU CHOSE OPTION 1:

Please ‘drag and drop’ each of the following reasons into the category that best reflects how important it was in making your decision. Multiple usage of the individual fields is possible.

Reason 1	There is scientific evidence that radiotherapy will benefit the patient.
Reason 2	I have had positive experiences with radiotherapy in dogs.
Reason 3	The client wants to try anything that might benefit the dog.
Reason 4	I think that every possible treatment option should be tried.

**Very important reason**	**Important reason**	**Less important reason**	**Not important at all**	**I don’t know**

IF YOU CHOSE OPTION 2:

Please 'drag and drop' each of the following reasons into the category that best reflects how important it was in making your decision. Multiple usage of the individual fields is possible.

Reason 1	I am not aware of scientific evidence that radiotherapy will benefit the patient.
Reason 2	I do not believe that radiotherapy will extend the dog's life.
Reason 3	The dog's age.
Reason 4	I think that dogs do not tolerate the side-effects.
Reason 5	The practice/clinic/hospital where I work does not offer radiotherapy.

**Very important reason**	**Important reason**	**Less important reason**	**Not important at all**	**I don't know**

IF YOU CHOSE OPTION 3:

Please 'drag and drop' each of the following reasons into the category that best reflects how important it was in making your decision. Multiple usage of the individual fields is possible.

Reason 1	I never answer the question "What would you do, if it was your animal?"
Reason 2	It is my responsibility to discuss all possible treatment options and side-effects, but not to decide for the client.

**Very important reason**	**Important reason**	**Less important reason**	**Not important at all**	**I don't know**

1.3: Cancer treatment: Cat with *feline injection-site sarcoma* (GROUP 2.a | other specialists and generalists)

An owner consults you about her twelve-year old cat that has developed a mass on the back of the neck four months after an annual vaccination. You suspect an injection site sarcoma. Staging shows no evidence of metastatic disease and radical surgical excision was performed. Histopathology confirmed the diagnosis, but has identified dirty margins. You discuss the option of referral to an oncologist for radiotherapy. Radiotherapy would reduce the risk of recurrence, but the client is unsure and asks you: “What would you do, if it was your cat?”

How would you advise your client? *(single choice)*

1	I would recommend radiotherapy and refer the patient to an oncologist.	
2	I would recommend against radiotherapy.	
3	I would not make any recommendation regarding radiotherapy.	
4	Other	

SUB-QUESTIONS:

IF YOU CHOSE OPTION 1:

Please ‘drag and drop’ each of the following reasons into the category that best reflects how important it was in making your decision. Multiple usage of the individual fields is possible.

Reason 1	There is scientific evidence that radiotherapy will benefit the patient.
Reason 2	I have had positive experiences with radiotherapy in cats.
Reason 3	The client wants to try anything that might benefit the cat.
Reason 4	I think that every possible treatment option should be tried.
Reason 5	The oncologist should be responsible for the final decision on radiotherapy.

Very important reason	Important reason	Less important reason	Not important at all	I don’t know

IF YOU CHOSE OPTION 2:

Please ‘drag and drop’ each of the following reasons into the category that best reflects how important it was in making your decision. Multiple usage of the individual fields is possible.

Reason 1	I am not aware of scientific evidence that radiotherapy will benefit the patient.
Reason 2	I do not believe that radiotherapy will extend the cat’s life.
Reason 3	The cat’s age.
Reason 4	I think that cats do not tolerate the side-effects.
Reason 5	There is no clinic offering this therapy nearby.

**Very important reason**	**Important reason**	**Less important reason**	**Not important at all**	**I don’t know**

IF YOU CHOSE OPTION 3:

Please ‘drag and drop’ each of the following reasons into the category that best reflects how important it was in making your decision. Multiple usage of the individual fields is possible.

Reason 1	I never answer the question “What would you do, if it was your animal?”
Reason 2	It is my responsibility to discuss all possible treatment options and side-effects, but not to decide for the client.
Reason 3	I do not have the expertise to make any recommendation.

**Very important reason**	**Important reason**	**Less important reason**	**Not important at all**	**I don’t know**

1.4: Cancer treatment: Dog with *a soft tissue sarcoma* (GROUP 2.b | other specialists and generalists)

An owner consults you about her twelve-year old mixed-breed dog that has developed a firm mass on the back of the neck. You suspect a soft tissue sarcoma (STS). Staging shows no evidence of metastatic disease and radical surgical excision was performed. Histopathology confirmed STS grade III with dirty margins. You discuss the option of referral to an oncologist for radiotherapy. Radiotherapy would reduce the risk of recurrence, but the client is unsure and asks you: “What would you do, if it was your dog?”

How would you advise the client? *(single choice)*

1	I would recommend radiotherapy and refer the patient to the oncologist.	
2	I would recommend against radiotherapy.	
3	I would not make any recommendation regarding radiotherapy.	
4	Other	

SUB-QUESTIONS:

IF YOU CHOSE OPTION 1:

Please ‘drag and drop’ each of the following reasons into the category that best reflects how important it was in making your decision. Multiple usage of the individual fields is possible.

Reason 1	There is scientific evidence that radiotherapy will benefit the patient.
Reason 2	I have had positive experiences with radiotherapy in dogs.
Reason 3	The client wants to try anything that might benefit the dog.
Reason 4	I think that every possible treatment option should tried.
Reason 5	The oncologist should be responsible for the final decision on radiotherapy.

**Very important reason**	**Important reason**	**Less important reason**	**Not important at all**	**I don’t know**

IF YOU CHOSE OPTION 2:

Please ‘drag and drop’ each of the following reasons into the category that best reflects how important it was in making your decision. Multiple usage of the individual fields is possible.

Reason 1	I am not aware of scientific evidence that radiotherapy will benefit the patient.
Reason 2	I do not believe that radiotherapy will extend the dog’s life.
Reason 3	The dog’s age.
Reason 4	I think that dogs do not tolerate the side-effects.
Reason 5	There is no clinic offering this therapy nearby.

**Very important reason**	**Important reason**	**Less important reason**	**Not important at all**	**I don’t know**

IF YOU CHOSE OPTION 3:

Please ‘drag and drop’ each of the reasons below into the category that best reflects how important it was in making your decision. Multiple usage of the individual fields is possible.

Reason 1	I never answer the question “What would you do, if it was your animal?”
Reason 2	It is my responsibility to discuss all possible treatment options and side-effects, but not to decide for the client.
Reason 3	I do not have the expertise to make any recommendation.

**Very important reason**	**Important reason**	**Less important reason**	**Not important at all**	**I don’t know**

C.2. Effect of client's different financial background on performing a CT scan to obtain a definitive diagnosis

(Experimental approach: The study population will be divided in three groups. Roughly thirty per cent of respondents will be faced with scenario 2.1, one third with scenario 2.2 and the last third with scenario 2.3)

Group 1.a, Group 2.a and Group 3.a *= all vets, who indicated to have a CT-scanner at the working place (Section A | Question 15) → this group of vets will be splitted in three different groups (different financial status of client)*

Group 1.b, Group 2.b and Group 3.b *= all vets, who indicated to have a NOT CT-scanner at the working place (Section A | Question 15) → this group of vets will be splitted in three different groups (different financial status of client)*

2.1: Client without health insurance but no financial limitations (GROUP 1.a – CT is available in your clinic/practice)

A client consults you about his 7-month-old Labrador, which has a moderately severe left foreleg lameness. The dog's elbow is painful on manipulation, and you suspect medial coronoid disease. You can take radiographs to exclude some types of elbow disease, but only a CT will enable a definitive diagnosis of coronoid disease to be made, and any fragments identified.

What would you do? *(single choice)*

1	I would suggest a CT scan.	
2	I would suggest conservative management (including analgesia) and re-assess the dog after several weeks.	
3	Other	

SUB-QUESTIONS:

IF YOU CHOOSE OPTION 1:

Please 'drag and drop' each of the following reasons into the category that best reflects how important it was in making your decision. Multiple usage of the individual fields is possible.

Reason 1	It is important to me to reach a definitive diagnosis.

Reason 2	Early diagnosis could enable more effective treatment to be offered.
Reason 3	I always offer every diagnostic option, but it is up to the client to decide.

**Very important reason**	**Important reason**	**Less important reason**	**Not important at all**	**I don't know**

IF YOU CHOOSE OPTION 2:

Please 'drag and drop' each of the following reasons into the category that best reflects how important it was in making your decision. Multiple usage of the individual fields is possible.

Reason 1	It is not important to me to reach a definitive diagnosis at this stage.
Reason 2	Before the dog has to be sedated for a CT-scan, I would see whether the dog will improve with conservative management.
Reason 3	Before the client has to spend money on a CT scan, I would see whether the dog will improve with conservative management.

**Very important reason**	**Important reason**	**Less important reason**	**Not important at all**	**I don't know**

2.2: Client without health insurance but no financial limitations (GROUP 1.b – CT is NOT available in the clinic/practice)

A client consults you about his 7-month-old Labrador, which has a moderately severe left foreleg lameness. The dog's elbow is painful on manipulation, and you suspect medial coronoid disease. You can take radiographs to exclude some types of elbow disease, but only a CT will enable a definitive diagnosis of coronoid disease to be made, and any fragments identified.

How would you proceed further? *(single choice)*

1	I would suggest a CT scan and refer the patient.	

2	I would suggest conservative management (including analgesia) and re-assess the dog after several weeks.	
3	Other	

SUB-QUESTIONS:

IF YOU CHOOSE OPTION 1:

Please 'drag and drop' each of the reasons below into the category that best reflects how important it was in making your decision. Multiple usage of the individual fields is possible.

Reason 1	It is important to me to reach a definitive diagnosis.
Reason 2	Early diagnosis could enable more effective treatment to be offered.
Reason 3	I always offer every diagnostic option, but it is up to the client to decide.

Very important reason	Important reason	Less important reason	Not important at all	I don't know

IF YOU CHOOSE OPTION 2:

Please 'drag and drop' each of the reasons below into the category that best reflects how important it was in making your decision. Multiple usage of the individual fields is possible.

Reason 1	It is not important to me to reach a definitive diagnosis at this stage.
Reason 2	Before the dog has to be sedated for a CT-scan, I would see whether the dog will improve with conservative management.
Reason 3	Before the client has to spend money on a CT scan, I would see whether the dog will improve with conservative management.
Reason 4	There is no clinic with a CT nearby.

Very important reason	Important reason	Less important reason	Not important at all	I don’t know

2.3: Client with health insurance (GROUP 2.a – CT is available in the clinic/practice)

A client consults you about his 7-month-old Labrador, which has a moderately severe left foreleg lameness. The dog’s elbow is painful on manipulation, and you suspect medial coronoid disease. You can take radiographs to exclude some types of elbow disease, but only a CT will enable a definitive diagnosis of coronoid disease to be made, and any fragments identified. The client tells you the dog is covered by health insurance.

What would you do? *(single choice)*

1	I would suggest a CT scan.	
2	I would suggest conservative management (including analgesia) and re-assess the dog after several weeks.	
3	Other	

SUB-QUESTIONS:

IF YOU CHOOSE OPTION 1:

Please ‘drag and drop’ each of the following reasons into the category that best reflects how important it was in your decision. Multiple usage of the individual fields is possible.

Reason 1	It is important to me to reach a definitive diagnosis.
Reason 2	Early diagnosis could enable more effective treatment to be offered.
Reason 3	I always offer every diagnostic option, but it is up to the client to decide.
Reason 4	The patient is covered by health insurance.

Very important reason	Important reason	Less important reason	Not important at all	I don't know

IF YOU CHOOSE OPTION 2:

Please 'drag and drop' each of the following reasons into the category that best reflects how important it was in making your decision. Multiple usage of the individual fields is possible.

Reason 1	It is not important to me to reach a definitive diagnosis at this stage.
Reason 2	Before the dog has to be sedated for a CT-scan, I would see whether the dog will improve with conservative management.
Reason 3	Before the client has to make use of the health insurance, I would see whether the dog will improve with conservative management.

Very important reason	Important reason	Less important reason	Not important at all	I don't know

2.4: Client with health insurance (GROUP 2.b – CT is NOT available in the clinic/practice)

A client consults you about his 7-month-old Labrador, which has a moderately severe left foreleg lameness. The dog's elbow is painful on manipulation, and you suspect medial coronoid disease. You can take radiographs to exclude some types of elbow disease, but only a CT will enable a definitive diagnosis of coronoid disease to be made, and any fragments identified. The client tells you the dog is covered by health insurance.

How would you proceed further? *(single choice)*

1	I would suggest a CT scan and refer the patient.	

2	I would suggest conservative management (including analgesia) and re-assess the dog after several weeks.	
3	Other	

SUB-QUESTIONS:

IF YOU CHOOSE OPTION 1:

Please 'drag and drop' each of the reasons below into the category that best reflects how important it was in making your decision. Multiple usage of the individual fields is possible.

Reason 1	It is important to me to reach a definitive diagnosis.
Reason 2	Early diagnosis could enable more effective treatment to be offered.
Reason 3	I always offer every diagnostic option, but it is up to the client to decide.
Reason 4	The patient is covered by health insurance.

Very important reason	Important reason	Less important reason	Not important at all	I don't know

IF YOU CHOOSE OPTION 2:

Please 'drag and drop' each of the following reasons into the category that best reflects how important it was in making your decision. Multiple usage of the individual fields is possible.

Reason 1	It is not important to me to reach a definitive diagnosis at this stage.
Reason 2	Before the dog has to be sedated for a CT-scan, I would see whether the dog will improve with conservative management.
Reason 3	Before the client has to make use of the health insurance, I would see whether the dog will improve with conservative management.

Reason 4	There is no clinic with a CT nearby.

Very important reason	Important reason	Less important reason	Not important at all	I don’t know

2.5: Client with financial limitations (GROUP 3.a – CT is available in the clinic/practice)

A client consults you about his 7-month-old Labrador, which has a moderately severe left foreleg lameness. The dog’s elbow is painful on manipulation, and you suspect medial coronoid disease. You can take radiographs to exclude some types of elbow disease, but only a CT will enable a definitive diagnosis of coronoid disease to be made, and any fragments identified. The client tells you that he has limited financial resources.

What would you do? *(single choice)*

1	I would suggest a CT scan.	
2	I would suggest conservative management (including analgesia) and re-assess the dog after several weeks.	
3	Other	

SUB-QUESTIONS:

IF YOU CHOOSE OPTION 1:

Please ‘drag and drop’ each of the following reasons into the category that best reflects how important it was in making your decision. Multiple usage of the individual fields is possible.

Reason 1	It is important to me to reach a definitive diagnosis.
Reason 2	Early diagnosis could enable more effective treatment to be offered.
Reason 3	I can offer an instalment payment plan to the client.
Reason 4	I always offer every diagnostic option, but it is up to the client to decide.

Very important resaon	Important reason	Less important reason	Not important at all	I don't know

IF YOU CHOOSE OPTION 2:

Please 'drag and drop' each of the following reasons into the category that best reflects how important it was in making your decision. Multiple usage of the individual fields is possible.

Reason 1	It is not important to me to reach a definitive diagnosis.
Reason 2	Before the dog has to be sedated for a CT-scan, I would see whether the dog will improve with conservative management.
Reason 3	I am not allowed to offer an instalment payment plan.
Reason 4	The client will not be able to afford arthroscopy / surgery, if indicated.
Reason 5	Due to economic aspects of the practice/clinic/hospital, I can only offer diagnostic investigations that the client can pay for.
Reason 6	It is important to consider the financial background of the client.

Very important reason	Important reason	Less important reason	Not important at all	I don't know

2.6: Client with financial limitations (GROUP 3.b – CT is NOT available in the clinic/practice)

A client consults you about his 7-month-old Labrador, which has a moderately severe left foreleg lameness. The dog's elbow is painful on manipulation, and you suspect medial coronoid disease. You can take radiographs to exclude some types of elbow disease, but only a CT will enable a definitive diagnosis of coronoid disease to be made, and any fragments identified. The client tells you that he has limited financial resources.

What would you do? *(single choice)*

1	I would suggest a CT scan and refer the patient.	
2	I would suggest conservative management (including analgesia) and re-assess the dog after several weeks.	
3	Other	

SUB-QUESTIONS:

IF YOU CHOOSE OPTION 1:

Please 'drag and drop' each of the following reasons into the category that best reflects how important it was in making your decision. Multiple usage of the individual fields is possible.

Reason 1	It is important to me to reach a definitive diagnosis.
Reason 2	Early diagnosis could enable more effective treatment to be offered.
Reason 3	I always offer every diagnostic option, but it is up to the client to decide.
Reason 4	I will try to find a referral clinic that will allow the client to pay in instalments.

**Very important reason**	**Important reason**	**Less important reason**	**Not important at all**	**I don't know**

IF YOU CHOOSE OPTION 2:

Please 'drag and drop' each of the following reasons into the category that best reflects how important it was in making your decision. Multiple usage of the individual fields is possible.

Reason 1	It is not important for me to reach a definitive diagnosis at this stage.
Reason 2	Before the dog has to be sedated for a CT-scan, I would see whether the dog will improve with conservative management.

Reason 3	The client will not be able to afford arthroscopy / surgery, if indicated.
Reason 4	There is no clinic with a CT nearby.
Reason 5	It is important to consider the financial background of the client.

Very important resaon	Important reason	Less important reason	Not important at all	I don't know

Thank you for participating in the survey!

If you would like to receive a short summary of the results, please click here to give your e-mail address.

You will will be forwarded to another "survey" to provide your address so that y**our address is not linked to your responses to this questionnaire.**

Please enter your e-mail address below to get informed about the results of the study in form of a short summary.
