# Supplementary material for: Managing conflicting ethical concerns in modern small animal practice—A comparative study of veterinarian’s decision ethics in Austria, Denmark and the UK
Source: PLoS One. 2021 Jun 18;16(6):e0253420. doi: 10.1371/journal.pone.0253420 (PMC8213188; doi:10.1371/journal.pone.0253420)
Supplement: S1 Table — (PDF) [file pone.0253420.s002.pdf]

S1 Table:

Socio-demographic and practice-specific factors for the whole study population and each sub-population from Austria, Denmark and the UK

	All countries (N=615-648)	Austria (n=95-102)	Denmark (n=161-172)	United Kingdom (n=357-374)	Tests
**GENDER**					
Male	181 (28.1)	25 (24.8)	43 (25.0)	113 (30.5)	$\chi^2(2)=2.466$, $p=0.291$
Female	462 (71.9)	76 (75.2)	129 (75.0)	257 (69.5)	
**AGE (in years) | Mean±Std.**					
	44.7±12.5	45.8±9.3	45.5±12.2	44.0±13.4	
**AGE (in years) | Median [IQR]**					
	45 [33;54]	46 [38;53]	46 [33;55]	44 [32;54]	
**AGE (in years)**					
23-29	88 (14.3)	5 (5.3)	16 (9.9)	67 (18.7)	
30-39	144 (23.4)	21 (22.1)	40 (24.8)	83 (23.1)	$H(2)=5.430$, $p=0.066$
40-49	147 (23.9)	27 (28.4)	40 (24.8)	80 (22.3)	
50-59	162 (26.3)	38 (40.0)	42 (26.1)	82 (22.8)	
≥ 60	74 (12.0)	4 (4.2)	23 (14.3)	47 (13.1)	
**WORK EXPERIENCE (in years)**					
0.5-5	137 (21.3)	11 (11.0)	39 (22.9)	87 (23.3)	
6-10	69 (10.7)	14 (14.0)	19 (11.2)	36 (9.7)	$H(2)=0.967$, $p=0.617$
11-20	135 (21.0)	29 (29.0)	32 (18.8)	74 (19.8)	
≥21	302 (47.0)	46 (46.0)	80 (47.1)	176 (47.2)	
**BUSINESS TYPE**					$\chi^2(4)=139.912$, $p<0.001$
Independently owned	374 (57.7)	97 (95.1)	131 (76.2)	146 (39.0)	AT vs. DK: $\chi^2(1)=14.430$, $p<0.001^a$, $\phi=.236$
Corporate owned	218 (33.6)	2 (2.0)	28 (16.3)	188 (50.3)	AT vs. UK: $\chi^2(1)=91.324$, $p<0.001^a$, $\phi=.459$
Other[b] (incl. university and shelter)	56 (8.6)	3 (2.9)	13 (7.6)	40 (10.7)	DK vs. UK: $\chi^2(1)=65.459$, $p<0.001^a$, $\phi=.364$
**EMPLOYMENT STATUS**					$\chi^2(4)=113.079$, $p<0.001$
Self-employed	229 (35.4)	81 (79.4)	63 (36.6)	85 (22.8)	AT vs. DK: $\chi^2(1)=46.230$, $p<0.001^a$, $\phi=.415$
Employed	397 (61.4)	20 (19.6)	105 (61.0)	272 (72.9)	AT vs. UK: $\chi^2(1)=108.329$, $p<0.001^a$, $\phi=.486$
Other[b] (retired and unspecified)	21 (3.2)	1 (1.0)	4 (2.3)	16 (4.3)	DK vs. UK: $\chi^2(1)=10.577$, $p<0.001^a$, $\phi=.142$
**INVOLVEMENT DAILY MANAGEMENT**					$\chi^2(2)=38.877$, $p<0.001$, $\phi=0.245$
Yes	422 (65.2)	93 (91.2)	95 (55.2)	234 (62.7)	AT vs. DK: $\chi^2(1)=38.413$, $p<0.001^a$, $\phi=.374$
No	225 (34.8)	9 (8.8)	77 (44.8)	139 (37.3)	AT vs. UK: $\chi^2(1)=30.207$, $p<0.001^a$, $\phi=.252$<br>DK vs. UK: $\chi^2(1)=2.769$, $p=0.096$
**INCOME DEPENDENT ON INCOME OF THE PRACTICE/CLINIC**					$\chi^2(2)=49.238$, $p<0.001$, $\phi=0.276$
Yes	432 (66.8)	79 (78.2)	78 (45.3)	275 (73.5)	AT vs. DK: $\chi^2(1)=28.134$, $p<.001^a$, $\phi=.321$
No	215 (33.2)	22 (21.8)	94 (54.7)	99 (26.5)	AT vs. UK: $\chi^2(1)=0.921$, $p=0.337$<br>DK vs. UK: $\chi^2(1)=40.097$, $p<0.001^a$, $\phi=.274$
**PERMISSION TO DISCOUNT FEES**					$\chi^2(2)=75.039$, $p<0.001$, $\phi=0.341$
Yes	461 (71.5)	94 (92.2)	150 (87.7)	217 (58.3)	AT vs. DK: $\chi^2(1)=1.325$, $p=0.250$
No	184 (28.59)	8 (7.8)	21 (12.3)	155 (41.7)	AT vs. UK: $\chi^2(1)=40.589$, $p<0.001^a$, $\phi=0.293$<br>DK vs. UK: $\chi^2(1)=46.179$, $p<0.001^a$, $\phi=.292$
**POST-GRADUATE QUALIFCATION**					
More clinical[1]	79 (12.6)	7 (7.2)	41 (24.6)	31 (8.5)	$\chi^2(4)=43.949$, $p<0.001$
More academic and other[2]	195 (31.0)	44 (45.4)	30 (18.0)	121 (33.2)	AT vs. DK: $\chi^2(2)=27.726$, $p<0.001^a$, $\phi=.324$<br>AT vs. UK: $\chi^2(2)=4.985$, $p=0.083$
No[3]	355 (56.4)	46 (47.4)	96 (57.5)	213 (58.4)	DK vs. UK: $\chi^2(2)=31.155$, $p<0.001^a$, $\phi=.242$

Counts (percentage)

[a] Bonferroni correction was applied for multiple comparison between three countries and significant variables

[b] Answer option "Other" were excluded from bivariate statistics

[1]AT: Fachtierarzt, Diplomate (e.g. European or American College); DK: Fagdyrlæge/Øjenpanel dyrlæge/eller anden tilsvarende efter-videreuddannelse; Master i familiedyrsvidenskab, Dansk specialdyrlæge godkendt af Fødevarestyrelsen; EBVS eller ABVS Diplomat; UK: Diplomate (e.g. European or American College); Masters

[2]AT: Doctor of veterinary medicine, PhD, Masters, other; DK: PhD, other UK: PhD, other;

[3]AT+DK+UK: I did not undertake any further qualification
