# Supplementary material for: Managing conflicting ethical concerns in modern small animal practice—A comparative study of veterinarian’s decision ethics in Austria, Denmark and the UK
Source: PLoS One. 2021 Jun 18;16(6):e0253420. doi: 10.1371/journal.pone.0253420 (PMC8213188; doi:10.1371/journal.pone.0253420)
Supplement: S2 Table — (PDF) [file pone.0253420.s003.pdf]

## ***S2 Table:*** *Decision ethics orientations scores across socio-demographic and practice-specific factors by countries*

| | **Client-empathetic** | | | **Development-oriented** | | | **Patient-focused** | | | **Client-devolved** | | |
|---|---|---|---|---|---|---|---|---|---|---|---|---|
| | Austria | Denmark | UK | Austria | Denmark | UK | Austria | Denmark | UK | Austria | Denmark | UK |
| **GENDER** | | | | | | | | | | | | |
| Male | 5.45±1.16 | 6.00±0.79 | 6.03±0.78 | 5.05±0.90 | 5.32±1.18 | 4.84±0.95 | 5.40±0.646 | 5.38±0.98 | 5.45±0.88 | 4.39±1.07 | 4.98±1.14 | 4.72±1.19 |
| Female | 5.48±1.08 | 6.18±0.61 | 6.03±0.75 | 5.21±1.03 | 5.32±0.08 | 4.48±0.96 | 5.56±0.842 | 5.50±0.90 | 5.37±0.86 | 4.45±1.27 | 4.87±0.94 | 5.00±0.96 |
| *p-value*[*] | t(99)=-0.124, p=0.901 | t(170)=-1.595, p=0.113 | t(386)=-0.061, p=0.951 | t(99)=-0.663, p=0.509 | t(99)=0.000, p=1.00 | t(368)=3.210, **p=0.001** | t(99)=-0.876, p=0.383 | t(170)=-0.759, p=0.449 | t(368)0.853, p=0.395 | t(99)=-0.231, p=0.818 | t(170)=0.606, p=0.545 | t(368)=-2.44, p=0.015 |
| **AGE *(in years)*** | | | | | | | | | | | | |
| 23-29 | 4.93±2.13 | 5.96±0.73 | 5.95±0.80 | 5.93±0.28 | 5.54±0.70 | 4.72±0.97 | 5.80±0.80 | 5.42±0.80 | 5.57±0.73 | 4.87±1.12 | 4.98±0.95 | 5.20±0.87 |
| 30-39 | 5.57±0.84 | 6.23±0.60 | 5.86±0.87 | 5.21±1.17 | 5.51±0.89 | 4.54±0.93 | 5.43±0.97 | 5.58±0.82 | 5.29±0.91 | 4.89±1.21 | 4.86±0.95 | 4.97±0.96 |
| 40-49 | 5.33±1.17 | 6.21±0.63 | 6.08±0.78 | 5.11±0.98 | 5.54±0.99 | 4.57±1.03 | 5.38±0.75 | 5.36±0.85 | 5.29±0.83 | 4.67±1.04 | 4.98±0.97 | 4.97±0.96 |
| 50-59 | 5.48±1.08 | 5.99±0.76 | 6.07±0.64 | 5.07±1.01 | 4.98±1.06 | 4.38±0.95 | 5.60±0.72 | 5.54±0.85 | 5.39±0.85 | 3.98±1.30 | 4.81±1.01 | 4.89±0.99 |
| ≥ 60 | 6.33±0.82 | 6.02±0.55 | 6.22±0.65 | 5.17±0.88 | 5.06±0.97 | 4.82±0.94 | 6.25±1.07 | 5.19±1.23 | 5.45±1.06 | 4.50±0.89 | 4.91±1.11 | 4.44±1.42 |
| *p-value*[**] | F(4,90)=1.029, p=0.397 | F(4,156)=1.177, p=0.323 | F(4,354)=2.078, p=0.083 | F(4,90)=0.826, p=0.512 | F(4,156)=2.413, p=0.051 | F(4,354)=1.972, p=0.098 | F(4,90)=1.315, p=0.510 | F(4,156)=0.868, p=0.458 | F(4,354)=1.331, p=0.258 | F(4,14.03)=2.061, p=0.140 | F(4,156)=0.186, p=0.946 | F(4,160.7)=2.962, **p=0.021, p=0.084**[a] |
| **WORK EXPERIENCE *(in years)*** | | | | | | | | | | | | |
| 0.5-5 | 5.36±1.54 | 6.08±0.64 | 5.97±0.86 | 5.79±0.72 | 5.56±0.88 | 4.72±0.98 | 5.64±0.86 | 5.43±0.70 | 5.55±0.70 | 4.45±1.04 | 4.87±0.89 | 5.22±0.85 |
| 6-10 | 5.76±0.59 | 6.26±0.63 | 5.69±0.94 | 4.79±1.22 | 5.23±0.85 | 4.42±0.94 | 5.10±0.96 | 5.84±0.99 | 5.30±1.08 | 5.12±1.19 | 4.86±0.96 | 4.89±0.93 |
| 11-20 | 5.18±1.18 | 6.16±0.69 | 6.09±0.67 | 5.21±0.97 | 5.35±1.12 | 4.59±0.95 | 5.59±0.72 | 5.40±0.82 | 5.32±0.87 | 4.6±1.14 | 4.84±1.12 | 4.95±0.99 |
| ≥21 | 5.59±1.02 | 6.11±0.67 | 6.10±0.68 | 5.04±0.95 | 5.21±0.99 | 4.52±0.98 | 5.59±0.76 | 5.43±1.03 | 5.37±0.89 | 4.12±1.22 | 4.94±1.01 | 4.76±1.15 |
| *p-value*[**] | F(3,96)=1.228, p=0.304 | F(3,166)=0.375, p=0.771 | F(3,117.3)=2.452, p=0.067 | F(3,96)=2.435, p=0.069 | F(3,166)=1.168, p=0.323 | F(3,369)=1.137, p=0.334 | F(3,96)=1.594, p=0.196 | F(3,166)=1.187, p=0.316 | F(3,369)=1.287, p=0.186 | F(3,32.01)=2.756, p=0.058 | F(3,166)=0.091, p=0.965 | F(3,128.80)=4.451, **p=0.005**[A], **p=0.015**[a] |
| **BUSINESS TYPE** | | | | | | | | | | | | |
| Independently | 5.47±1.10 | 6.14±0.72 | 6.08±0.67 | 5.16±0.99 | 5.33±0.95 | 4.60±0.97 | 5.52±0.79 | 5.46±0.90 | 5.53±0.85 | 4.38±1.21 | 4.90±1.02 | 4.87±1.12 |
| Corporate | 4.67±0.47 | 6.08±0.53 | 6.00±0.83 | 4.17±1.18 | 5.13±1.11 | 4.63±0.98 | 5.83±1.18 | 5.24±0.97 | 5.31±0.88 | 4.50±0.24 | 4.94±0.86 | 5.05±0.97 |
| Other (incl. uni and shelter) | 1.00±0.67 | 6.15±0.48 | 5.93±0.74 | 5.67±0.89 | 5.61±0.92 | 4.27±0.89 | 5.33±1.15 | 6.00±0.83 | 5.27±0.82 | 5.89±0.19 | 4.74±1.06 | 4.48±0.94 |
| *p-value*[*]*(other excluded from t-test)* | t(97)=1.028, p=0.307 | t(157)=0.429, p=0.668 | t(332)=0.865, p=0.388 | t(97)=1.398, p=0.165 | t(157)=0.966, p=0.355 | t(332)=-0.258, p=0.797 | t(97)=-0.556, p=0.579 | t(157)=1.191, p=0.235 | t(332)=2.349, **p=0.019** | t(97)=-0.137, p=0.891 | t(157)=-0.180, p=0.857 | t(332)=-1.600, p=0.111 |
| **EMPLOYMENT TYPE** | | | | | | | | | | | | |
| Self-employed | 5.45±1.05 | 6.12±0.76 | 5.95±0.86 | 5.05±0.96 | 5.30±0.93 | 4.60±0.98 | 5.53±0.71 | 5.49±0.96 | 5.39±0.99 | 4.28±1.23 | 4.84±1.07 | 4.82±1.16 |
| Employed | 5.50±1.27 | 6.14±0.60 | 6.04±0.74 | 5.52±1.08 | 5.30±1.00 | 4.57±0.98 | 5.40±1.04 | 5.45±0.90 | 5.40±0.82 | 4.98±1.01 | 4.91±0.95 | 4.96±1.00 |
| *p-value* | t(99)=-0.166, p=0.869 | t(166)=-0.114, p=0.909 | t(355)=-0.934, p=0.351 | t(99)=-1.886, p=0.062 | t(166)=0.000, p=1.00 | t(355)=0.299, p=0.765 | t(99)=0.667, p=0.507 | t(166)=0.270, p=0.787 | t(355)=-0.123, p=0.902 | t(99)=-2.373, **p=0.020** | t(166)=-0.493, p=0.623 | t(355)=-1.036, p=0.301 |
| **POST-GRADUATE QUALIFCATION** | | | | | | | | | | | | |
| Clinical[1] | 5.62±1.18 | 6.02±0.69 | 6.01±0.88 | 5.19±1.33 | 5.28±1.15 | 4.58±1.11 | 5.76±0.71 | 5.61±1.03 | 5.44±0.79 | 3.67±0.96 | 5.24±0.93 | 4.84±1.25 |
| Academic[2] | 5.42±1.00 | 6.33±0.09 | 6.15±0.65 | 5.11±0.77 | 5.40±1.06 | 4.69±0.94 | 5.58±0.71 | 5.54±0.82 | 5.40±0.89 | 4.47±1.20 | 4.59±1.11 | 4.83±1.01 |
| No[3] | 5.58±1.17 | 6.10±0.68 | 5.96±0.78 | 5.18±1.16 | 5.26±0.86 | 4.50±0.95 | 5.48±0.82 | 5.39±0.89 | 5.37±0.88 | 4.59±1.25 | 4.89±0.95 | 4.97±1.03 |
| *p-value*[**] | F(2,94)=0.275, p=0.760 | F(2,164)=2.073, p=0.129 | F(2,362)=2.445, p=0.088 | F(2,94)=0.055, p=0.946 | F(2,164)=0.239, p=0.787 | F(2,362)=1.470, p=0.231 | F(2,94)=0.510, p=0.602 | F(2,164)=0.928, p=0.398 | F(2,362)=0.104, p=0.901 | F(2,94)=1.791, p=0.172 | F(2,65.135)=3.669, p=0.031, p=0.062[a] | F(2,362)=0.741, p=0.477 |

*[*]from independent t-test; [**]from F-test; Post hoc analysis: [A] Client-devolved UK: "0.5-5 years experiences" vs. "≥20 years experiences": d=0.45, p=.002; [1]AT: Fachtierarzt, Diplomate; DK: Fagdyrlæge/Øjenpanel dyrlæge/eller anden tilsvarende efter-videreuddannelse; Master i familiedyrsvidenskab, Dansk specialdyrlæge godkendt af Fødevarestyrelsen; EBVS eller ABVS Diplomat; UK: Diploma (e.g. RCVS, European or American College); Masters; [2]AT: Doctor of veterinary medicine, PhD, Masters, other; DK: PhD, other UK: PhD, other; [3]AT+DK+UK: I did not undertake any further qualification; [a]p-values after Bonferroni correction*
